# The Efficacy of Be a Mom, a Web-Based Intervention to Prevent Postpartum Depression: Examining Mechanisms of Change in a Randomized Controlled Trial

**DOI:** 10.2196/39253

**Published:** 2023-03-17

**Authors:** Carlos Carona, Marco Pereira, Anabela Araújo-Pedrosa, Maria Cristina Canavarro, Ana Fonseca

**Affiliations:** 1 Center for Research in Neuropsychology and Cognitive-Behavioral Intervention University of Coimbra Coimbra Portugal; 2 Obstetrics Unit A Centro Hospitalar e Universitário de Coimbra Coimbra Portugal

**Keywords:** Be a Mom, randomized controlled trial, postpartum depression, web-based interventions, cognitive behavioral therapy, prevention, mobile phone

## Abstract

**Background:**

Postpartum depression (PPD) is treatable and preventable, but most women do not seek professional help for their perinatal depressive symptoms. One increasingly popular approach of improving access to care is the use of web-based intervention programs.

**Objective:**

The objective of this study was 2-fold: first, to assess the efficacy of *Be a Mom*, a brief web-based selective or indicated preventive intervention, in reducing depressive and anxiety symptoms of women at high risk for PPD; and second, to examine mechanisms of change linking modifiable self-regulatory skills (ie, emotion regulation, self-compassion, and psychological flexibility) to improved perinatal mental health outcomes.

**Methods:**

This 2-arm, open-label randomized controlled trial involved a sample of 1053 perinatal women presenting high risk for PPD who were allocated to the *Be a Mom* intervention group or a waitlist control group and completed self-report measures at baseline and postintervention assessments. Univariate latent change score models were computed to determine changes over time in adjustment processes and outcomes, with a multigroup-model approach to detect differences between the intervention and control groups and a 2-wave latent change score model to examine whether changes in processes were related to changes in outcomes.

**Results:**

*Be a Mom* was found to be effective in reducing depressive (intervention group: µΔ=–3.35; *P*<.001 vs control group: µΔ=–1.48; *P*<.001) and anxiety symptoms (intervention group: µΔ=–2.24; *P*<.001 vs control group: µΔ=–0.43; *P*=.04) in comparison with the control group, where such changes were inexistent or much smaller. All 3 psychological processes under study improved statistically significantly in posttreatment assessments: emotion regulation ability (Δχ^2^_3_=12.3; *P*=.007) and psychological flexibility (Δχ^2^_3_=34.9; *P*<.001) improved only in the intervention group, and although self-compassion increased in both groups (Δχ^2^_3_=65.6; *P*<.001), these improvements were considerably greater in the intervention group.

**Conclusions:**

These results suggest that *Be a Mom*, a low-intensity cognitive behavioral therapy program, is a promising first-line intervention for helping perinatal women, particularly those with early-onset PPD symptoms.

**Trial Registration:**

ClinicalTrials.gov NCT03024645; https://clinicaltrials.gov/ct2/show/NCT03024645

## Introduction

### Background

The perinatal period is a time of physical, psychological, and interpersonal changes, representing a particularly vulnerable moment in a woman’s life for the development of mental health problems [1]. During this challenging period, marked by positive and negative emotions that vary in frequency and intensity over time, women are at increased risk of developing mental health conditions of differing levels of severity, ranging from mild and transient emotional disturbance to more severe forms of psychopathology [2]. Postpartum depression (PPD), often concurrent with heightened anxiety, is a common and serious perinatal disorder with devastating and enduring effects on mothers’ well-being, infants’ health development, marital relationship, and overall family functioning [3].

Although PPD is treatable and amenable to preventive efforts, most women do not seek professional help for their perinatal depression symptoms [4], with recent research showing that attitudinal (eg, thinking that no one will be able to help), knowledge (eg, not knowing whether one’s problems are a reason to ask for help) and structural barriers (eg, not having time or not being able to afford treatment) are the most common help-seeking barriers of perinatal women [5]. One increasingly popular approach of improving access to treatment is the use of web-based intervention programs, particularly low-intensity interventions. These interventions offer several advantages over traditional formats of assistance, including greater temporal and local independency, anonymity, accessibility, and flexible delivery [6], making them particularly recommended for perinatal women.

Other web-based interventions have been developed for PPD [7], but most of them were not designed or tested for preventive purposes (with the notable exceptions of *Mothers and Babies Course* [8] and *Sunnyside* [9], which nevertheless failed to provide compelling evidence on their efficacy), and they relied exclusively on classic cognitive behavioral therapy (CBT) principles [10,11], thus neglecting various concepts emphasized by *third wave* CBT (eg, acceptance and cognitive defusion), which were recently added to traditional CBT packages to foster intervention processes and outcomes [12]. *Third wave* CBT represents an evolution and extension of traditional CBT approaches by highlighting the role of changeable transdiagnostic psychological processes (ie, functionally important pathways of change) over the reduction or elimination of psychological and emotional symptoms. *Third wave* methods emphasized issues such as emotion regulation, acceptance, values, goals, and metacognition, and rather than focusing on the content of a person’s thoughts and internal experiences, they focused on the context, processes, and functions of how a person relates to their internal experiences [13].

### *Be a Mom*, a Web-Based Intervention to Prevent PPD

*Be a Mom* is a brief, self-guided, web-based selective or indicated preventive intervention that is primarily targeted at women who exhibit risk for PPD or present early-onset PPD symptoms. Grounded in CBT principles, it combines the classic CBT approach (eg, cognitive restructuring and activity scheduling) with more recent third wave CBT features (eg, values clarification and self-compassion). *Be a Mom* is a structured program with a modular approach that encompasses five sequential modules on the following topics: (1) changes and emotional reactions (changes and reorganizations during transition to parenthood; unrealistic expectations, role idealization, and the myths of perfect motherhood; and the cognitive-emotional-behavioral link); (2) cognitions (negative thoughts; reducing the power of thoughts: questioning and defusing; and self-criticism and self-compassion); (3) values and social support (values and commitment; social network: how to identify support needs and ask for help; and assertive communication: dealing with family and friends); (4) couple relationship (changes in the couple relationship during the postpartum period; assertive communication within the couple: negotiation and conflict resolution skills; and sharing parenthood values and commitments); and (5) PPD alert signs and professional help seeking (identify PPD signs and symptoms; professional help seeking: treatment options and how to seek help; and a continuing journey: planning for the future). The content of each module includes psychoeducational information combined with practical exercises and endorses the structured and goal-oriented nature of CBT sessions [14].

Preliminary evidence from a pilot randomized controlled trial (RCT) attested the feasibility of the program and its effectiveness in reducing depressive and anxiety symptoms of women at high risk for PPD [14]. Furthermore, a greater decrease in depressive symptoms was found to be associated with a greater decrease in emotion regulation difficulties and a greater increase in self-compassion, whereas psychological flexibility was found to be unrelated to changes in depression levels [15].

### This Study

**Figure 1:**
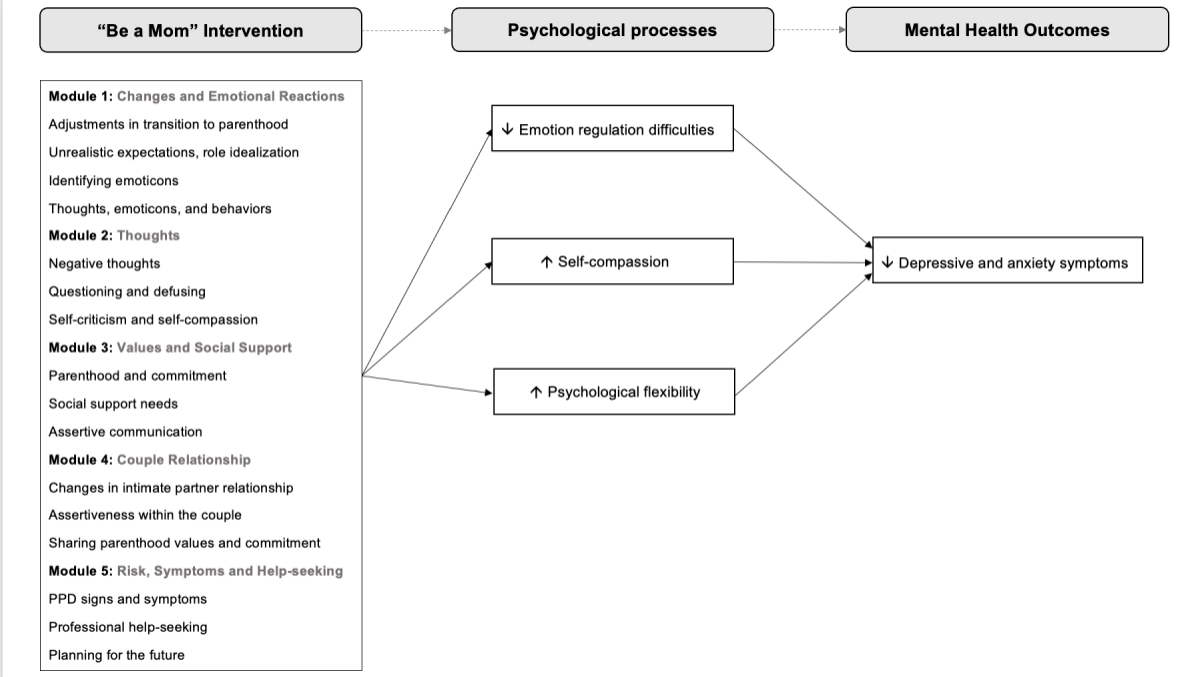
Conceptual diagram of the study.

Although the mechanisms of change are the core of evidence-based interventions [12], an examination of these mechanisms within the context of web-based interventions for PPD had not been performed until recently [15]. Given the fact that interventions to prevent PPD are the most effective when they target women considered to be at risk [16], this RCT focused on a large sample of women at high risk for PPD to ascertain the mechanisms of change associated with the efficacy of *Be a Mom* in reducing PPD and postpartum anxiety symptoms because they often occur in comorbidity. Specifically, bearing in mind that depression and anxiety are distinct but highly concurrent clinical phenomena [3], the study sought to examine whether changes in psychological processes (ie, emotion regulation, self-compassion, and psychological flexibility) were associated with changes in depressive and anxiety symptoms among women who participated in the *Be a Mom* program (Figure 1).

## Methods

### Procedure

This study was a 2-arm, open-label RCT to assess the efficacy of the *Be a Mom* intervention, in comparison with a waitlist control group receiving usual care, among women presenting high risk for PPD. The trial has been registered at ClinicalTrials.gov (NCT03024645). The eligibility criteria to participate in the study were as follows: (1) being an adult woman (aged ≥18 years) in the early postpartum period (up to 3 months post partum), (2) presenting high risk for PPD (a score of ≥5.5 on the Postpartum Depression Predictors Inventory-Revised [PDPI-R; refer to the *Measures* subsection]), (3) having a computer or tablet device or smartphone and internet access at home, (4) having the ability to read and speak Portuguese, and (5) being a resident of Portugal. The exclusion criterion was the presence of a severe medically diagnosed health condition in the infant or in the mother (eg, cerebral palsy in the infant or schizophrenia spectrum or other psychotic disorders in the mother), as self-reported by mothers. The women who did not meet the eligibility criteria were informed through email and were advised to seek professional help, if needed.

Recruitment occurred on the web between January 25, 2019, and January 30, 2021, both through unpaid cross-posting and paid advertisements on social media networks (Facebook and Instagram). Paid advertisements and campaigns targeted women aged 18 to 45 years with interests in maternity topics with the following tagline: “Did you have a baby in the last three months? We want to know if ‘Be a Mom’ is effective in promoting postpartum women’s mental health, and you can help us! To know if you are eligible to participate in the study fill out the following form and we will contact you.” Before receiving access to the eligibility form (including a set of questions to assess eligibility criteria and contact information), women were given information about the study goals and procedures (including voluntary participation and data protection issues), the participants’ and researchers’ roles were clarified, and women were asked to provide their informed consent to participate in the study (by clicking the option “I understand and accept the conditions of the study”).

Eligible women who consented to participate in the study were administered baseline assessment (T0) using the web-based survey platform LimeSurvey (LimeSurvey GmbH). After completing the baseline assessment, eligible participants were randomly assigned using a computerized random number generator (allocation rate: 1:1) to the intervention group (*Be a Mom* intervention) or to the control group (waitlist group receiving usual care). According to the guidelines established by the pregnancy surveillance program in Portugal [17], usual postpartum care encompasses at least 1 medical appointment with a designated obstetrician or the woman’s family physician, as well as weekly visits to nursing services in primary care to monitor the general health status of the mothers and their infants during the first 2 months post partum. One of the researchers was responsible for randomization, whereas the other 2 researchers were responsible for the participants’ enrollment and group assignment. Participants were informed about their assigned group through email (no blinding to the assigned group) and instructed to seek no other intervention besides *Be a Mom* or usual care while participating in the RCT. Postintervention (T1) assessment was also performed on the web (through the LimeSurvey platform) in both intervention and control groups (after the intervention or 8 weeks after randomization). To reduce attrition, weekly reminders (email and SMS text messages on an alternate basis) were sent during 1 month to women who failed to complete the baseline and postintervention assessments.

The CONSORT-eHEALTH (Consolidated Standards of Reporting Trials of Electronic and Mobile Health Applications and Online Telehealth) checklist [18,19] (See Multimedia Appendix 1) was used for study reporting.

### Intervention and Control Arms

Women assigned to the intervention arm were invited by email to register on a password-protected website that contained the *Be a Mom* intervention [20] (access to the program was restricted to the invited women). Approximately 2 weeks after registration, participants were contacted via telephone by the research team to assess whether there were any difficulties in accessing the website and to clarify any questions concerning the program’s flow and functioning. After registration, the women had access to the 5 modules (the *Couple Relationship* module was only presented to women in a relationship), and they were instructed to complete 1 module per week in sequence, although a slower pace was allowed. Each module is approximately 45 minutes long. The women were given the option of pausing the module and resuming a session from the last page visited during subsequent access. Although it was generally recommended that they complete 1 module per week, given the usual competing demands of the postpartum period, they were allowed to complete the program within a maximum period of 8 weeks. Email reminders were sent automatically to the participants if they went 3, 7, and 13 days without accessing the program. The periodicity of the reminders was defined according to the guidelines provided by the software company that developed the web-based program and based on previous feedback obtained from postpartum women during the pilot RCT [14]. Of note, the sequence of periodical reminders restarted every time a woman returned to proceed with the program, that is, the reminders were unrelated to the amount of content that had already been completed. Asynchronous communication channels were provided for program-related support only. Access to the program was free of cost, and no compensation was offered to participants. Participants assigned to the control group were offered no intervention but were free to access usual care (as were all participants). At the end of the RCT (including 3-month and 12-month follow-up assessments), they were offered access to the *Be a Mom* intervention. Given that the first 3 to 4 months post partum seem to be a high-risk period for depression [21], the women in the waitlist control group were informed at the outset about their risk for PPD and instructed to interrupt their participation in the RCT and seek professional help within usual care if they felt that their health condition was worsening.

### Measures

#### Sociodemographic and Clinical Information

A self-report form was used to gather information concerning the women’s sociodemographic (eg, age, marital status, number of children, employment status, educational level, household monthly income, and residence), clinical (eg, risk score), and infant (eg, age and sex) data.

#### PPD Risk

The Portuguese version of the PDPI-R [22] was used to identify women presenting high risk for PPD. This self-report questionnaire includes 39 items assessing well-identified risk factors for PPD (eg, prenatal depression and low social support) answered on a *yes* versus *no* dichotomous scale (except for items assessing marital and socioeconomic status). The PDPI-R total score ranges from 0 to 39, with higher scores indicating an increased risk for PPD. In Portuguese validation studies, a score of ≥5.5 was indicative of higher risk for PPD [22].

#### Adjustment Outcomes

##### Depressive Symptoms

The Portuguese version of the Edinburgh Postpartum Depression Scale (EPDS) [23] was used to assess PPD symptoms. The EPDS comprises 10 items that assess symptoms of depression in terms of their presence and severity in the previous 7 days. The items were rated on a 4-point frequency scale (ranging from 0 to 3), with higher scores being indicative of more severe depressive symptoms. In this study, the Cronbach α values for the EPDS were ≥.86.

##### Anxiety Symptoms

The anxiety subscale of the Hospital Anxiety and Depression Scale (Portuguese version) was used to assess anxiety symptoms [24]. The anxiety subscale comprises 7 items, answered on a 4-point response scale (ranging from 0 to 3) to evaluate the presence of anxiety symptoms in the week before completion of the program. Higher scores were indicative of more severe anxiety symptoms. The Cronbach α values for the anxiety subscale were ≥.82.

#### Psychological Processes

##### Emotion Regulation

The Portuguese version of the Difficulties in Emotion Regulation Scale-Short Form [25] was used to assess women’s emotion regulation abilities. This self-report questionnaire comprises 18 items developed to assess difficulties in using adaptive emotional regulation strategies, which were answered on a 5-point scale (ranging from 1=*almost never* to 5=*almost always*). The total score is used as a measure of emotion dysregulation [17]. Higher scores were suggestive of lower emotion regulation ability. The Cronbach α values for this scale were ≥.91.

##### Self-compassion

The Portuguese version of the Self-Compassion Scale-Short Form (SCS-SF) [26] was used to assess the women’s levels of self-compassion. The SCS-SF is a self-report questionnaire with 12 items, answered on a 5-point scale (ranging from 1=*almost never* to 5=*almost always*). A total score may be computed, with higher scores being indicative of higher levels of self-compassion. In this study, the Cronbach α values for the SCS-SF were ≥.88.

##### Psychological Flexibility

The Portuguese version of the Comprehensive Assessment of Acceptance and Commitment Therapy Processes (CompACT) [27] was used in this study to measure psychological flexibility. The CompACT is a self-report questionnaire with 18 items organized into 3 subscales: openness to experience, behavioral awareness, and valued action. The items were answered on a 7-point response scale (ranging from 0=*strongly disagree* to 6=*strongly disagree*). It is possible to compute a total score, with higher scores indicating higher psychological flexibility. In this study, the Cronbach α values for the CompACT were ≥.86.

### Data Analyses

SPSS software (version 22.0; IBM Corp) was used for descriptive and comparative analyses, and the Mplus program (version 7.0; Muthén & Muthén) was used for the examination of univariate latent change score (LCS) models. Descriptive statistics and comparison tests were computed for sample characterization and for comparing study completers and dropouts. Missed end points ranged from 32.4% (EPDS) to 40.2% (SCS-SF), and the pattern-of-missingness analysis suggested that data are missing completely at random (Little missing completely at random test: χ^2^_14_=9.7; *P*=.78). Missing data were handled using the full information maximum likelihood estimation [28].

**Figure 2:**
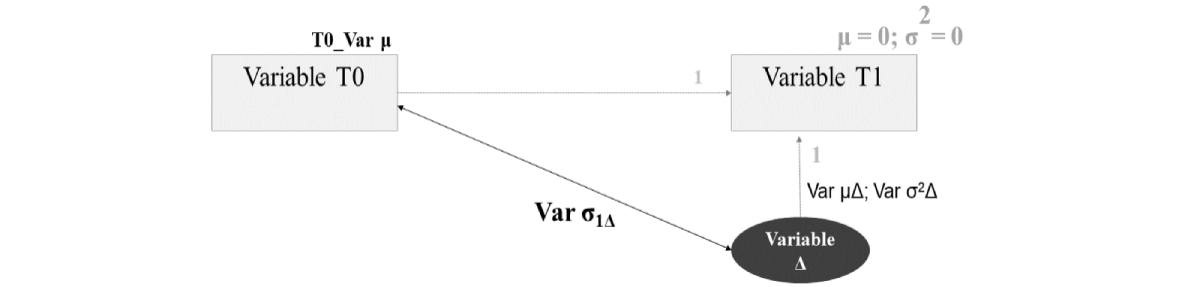
Univariate latent change score–model representation. Var: variable.

To examine changes over time both in the adjustment outcomes (depressive and anxiety symptoms) and in the psychological processes (emotion regulation, self-compassion, and psychological flexibility), univariate LCS models were computed [29]. LCS is a structural equation modeling approach in which a within-participants approach is considered, with change between 2 time points in 1 variable being modeled as a latent factor (defined as the part of the score of the variable at T1 that is not identical to the score of the variable at T0). LCS models allow the estimation of (1) the mean intercept of the change between T0 and T1 (µΔ, latent factor), (2) the variance or residual variance of the change between T0 and T1 (α^2^Δ), (3) the covariance between the individual’s score at T0 and the latent change factor (α1Δ), and (4) the mean scores at T0 (Figure 2). A statistically significant positive mean intercept of the change (µΔ) suggests that, on average, an individual’s scores increased over time, whereas a statistically significant negative mean intercept suggests a decrease in the individual’s scores from T0 to T1. Moreover, a statistically significant variance or residual variance in the LCS factor suggests heterogeneity across individuals regarding the averaged trajectory [29,30].

A multigroup-model approach was used to check for differences between the intervention and control groups in LCS models: the full constrained model (in which the LCS estimates were constrained to be equal across the groups) and the unconstrained model (in which the LCS estimates were free to vary across the groups) were compared concerning model fit indices and chi-square differences (Δχ^2^). Statistically significant chi-square changes (Δχ^2^) indicate that the LCS model is different across the groups. The overall model fit was ascertained using the chi-square goodness-of-fit statistic (significance at *P*>.05), the comparative fit index (CFI; ≥0.95), the root mean square of approximation (RMSEA; ≤0.08), and the standardized root mean square residual (SRMR; ≤0.08) [31,32]. In addition to the main analyses, multigroup-model comparison analyses (in which the full constrained model and the unconstrained models were compared) were repeated in 2 subgroups of women: evaluative respondents (ie, intention-to-treat analysis, by including women who completed both baseline and postintervention assessments in the statistical analyses) and women presenting clinically relevant depressive symptoms (ie, baseline EPDS scores of >9). Although *Be a Mom* was originally developed as a web-based CBT intervention to prevent PPD, the multigroup comparison analysis was conducted in this subgroup of women to ascertain the efficacy of *Be a Mom* in improving the mental health of those women who were already presenting clinical levels of depressive symptoms.

Finally, to examine whether changes in psychological processes (emotion regulation, self-compassion, and psychological flexibility) were associated with changes in women’s adjustment outcomes (depressive and anxiety symptoms), a 2-wave LCS (2W-LCS) model was tested [30,33,34]. The 2W-LCS allows the estimation of the univariate LCS for each variable (ie, psychological processes: emotion regulation, self-compassion, and psychological flexibility; and adjustment outcomes: depressive and anxiety symptoms) and of the change-to-change effects (ie, the estimate of the effect of the change in one variable on the change of the other variable) of psychological processes (emotion regulation, self-compassion, and psychological flexibility) on adjustment outcomes. A positive and significative regression coefficient indicates that higher change scores in the antecedent variable are associated with higher change scores in the outcome variable, whereas a negative and significative regression coefficient indicates that higher change scores in the antecedent variable are associated with lower change scores in the outcome variable. As this study adopted a 2–time point longitudinal design, 2W-LCS was preferred over mediational analyses that typically require a 3–time point longitudinal design to be accurately performed. Moreover, this study sought to examine whether the proposed mechanisms of change differed in the intervention and control groups across time before analyzing their associations with changes in the levels of depressive and anxiety symptoms.

The 2W-LCS model allows the estimation of the correlations among individuals’ scores at baseline (ie, correlations among emotion regulation, self-compassion, psychological flexibility, depressive symptoms, and anxiety symptoms) and the correlations among LCSs (ie, correlations among changes in emotion regulation, self-compassion, and psychological flexibility, as well as correlations between depressive and anxiety symptoms). In addition, the 2W-LCS model allows the estimation of the cross-lagged paths between the scores of psychological processes (emotion regulation, self-compassion, and psychological flexibility) at baseline and the change score of anxiety and depression symptoms, as well as that of the cross-lagged paths between the scores of depressive and anxiety symptoms at baseline and the change score of psychological processes. A multigroup-model approach was used to check for differences between the intervention and control groups in 2W-LCS models: the full constrained model (in which the 2W-LCS estimates were constrained to be equal across the groups) was compared with the unconstrained model concerning model fit indices and chi-square differences (Δχ^2^).

### Ethics Approval

The study was approved by the ethics committee of the Faculty of Psychology and Educational Sciences, University of Coimbra (FPCE-CEDI-17.01.19).

## Results

### Participants

**Figure 3:**
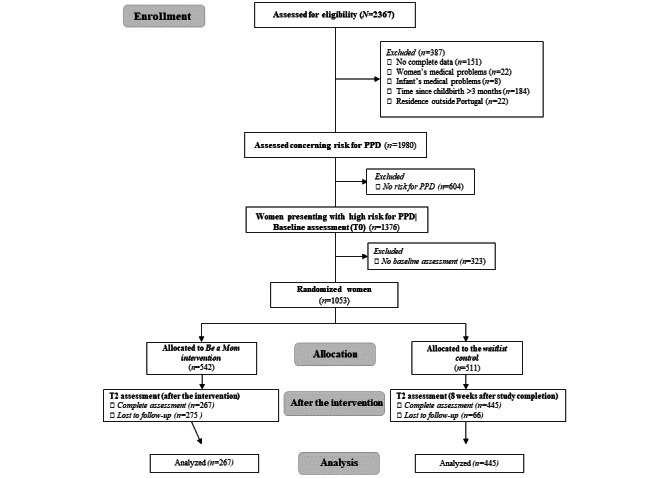
Participant flow diagram. PPD: postpartum depression.

Of the 2367 women enrolled in the study between January 25, 2019, and January 30, 2021, a total of 1980 (83.65%) women were assessed concerning risk for PPD. Of these 1980 women, 1376 (69.49%) who presented high risk for PPD were administered the baseline assessment. Of the 1376 eligible women, 1053 (76.52%) completed baseline assessment and were randomized to the intervention (n=542, 51.47%) or control (n=511, 48.52%) conditions. The participant flowchart is presented in Figure 3. The baseline sociodemographic and clinical characteristics of the participants are presented in Table 1. No baseline differences were found between the groups, with the exception of parity: a higher number of women in the intervention group reported that this is their first child (347/542, 63.7%, vs 296/511, 57.7%, in the control group; *P*=.046). Moreover, of the 1053 participants, 341 (32.38%) did not complete the postintervention assessments. Of the 267 intervention group participants who completed the postintervention assessments, 129 (48.3%) completed the *Be a Mom* program, whereas 62 (23.2%) only partially completed the program (ie, completed 2 or 3 modules), and 76 (28.5%) did not complete the program (ie, completed only 1 module). Differences between study completers and dropouts in terms of baseline characteristics were found concerning marital status (*χ*^2^_3_=14.2; *P*=.003; a higher proportion of the dropouts were not married or cohabiting: completers, 61/712, 8.6%, vs dropouts, 73/341, 15.6%), educational level (χ^2^_5_=19.7; *P*=.001; a higher proportion of the completers had a higher education degree: completers, 315/483, 65.2%, vs dropouts, 124/250, 49.5%), infant age (t_1051_=2.29; *P*=.02; women who dropped out from the study had older infants [mean age: 2.13, SD 0.98 years] than those who completed the study [mean age: 1.98, SD 0.94 years]), and level of risk (t_1051_=3.88; *P*<.001; women who dropped out from the study presented higher risk for PPD [mean 12.14, SD 5.00] than those who completed the study [mean 10.99, SD 4.26]). Of note, despite the higher proportion of dropouts in the intervention group than in the control group, the sociodemographic and clinical characteristics of women who completed both assessments (*evaluative respondents*) are similar in both groups, with the exception of parity (*P*<.001). Variables that differ between the intervention and control groups and between the completers and dropouts were introduced as covariates in the analyses.

**Table 1:** Baseline sociodemographic and clinical characteristics: comparison between the control and intervention groups (N=1053).

		Control group (n=511)	Intervention group (n=542)	Chi-square (*df*)	*t* test (*df*)	*P* value
**Women’s sociodemographic characteristics**						
	Age (years), mean (SD)	32.81 (4.66)	32.91 (4.40)	N/A^a^	–0.35 (1051)	.73
	Marital status (married or living together), n (%)	457 (89.4)	482 (88.9)	1.5 (3)	N/A	.69
	Number of children (primiparous), n (%)	296 (57.9)	347 (64.0)	4.0 (1)	N/A	.046
	Educational level (higher education), n (%)	214 (60.2)	225 (59.5)	3.5 (5)	N/A	.63
	Professional status (employed), n (%)	427 (85.3)	465 (88.1)	6.3 (4)	N/A	.18
	Monthly income (<€1000 [<US $1074]), n (%)	325 (63.6)	344 (63.4)	3.6 (4)	N/A	.46
	Residence (urban), n (%)	404 (79.1)	434 (80.1)	0.2 (1)	N/A	.68
Women’s baseline risk score (PDPI-R^b^), mean (SD)		11.44 (4.57)	11.28 (4.53)	N/A	0.57 (1051)	.57
**Infant’s characteristics**						
	Age (months), mean (SD)	1.99 (0.93)	2.06 (0.98)	N/A	–1.08 (1051)	.28
	Sex (male), n (%)	263 (51.5)	260 (48)	2.8 (1)	N/A	.25

^a^N/A: not applicable.

^b^PDPI-R: Postpartum Depression Predictors Inventory-Revised.

### Change From Baseline to Posttreatment Assessment in Adjustment Outcomes and Psychological Processes: Comparing the Intervention and Control Groups

Table 2 presents the descriptive statistics at the baseline and postintervention assessments for the study variables, as well as the LCS estimates for the intervention and control groups.

Concerning adjustment outcomes (depressive and anxiety symptoms), the full constrained model presented globally a poor fit to data (depressive symptoms: χ^2^_4_=41.4; *P*<.001; CFI=0.894; RMSEA=0.133, 90% CI 0.098-0.171; *P*<.001; SRMR=0.111; anxiety symptoms: χ^2^_4_=36.0; *P*<.001; CFI=0.906; RMSEA=0.123, 90% CI 0.088-0.162; *P*<.001; SRMR=0.097), whereas the unconstrained model presented a good fit (depressive symptoms: χ^2^_1_=5.8; *P*=.02; CFI=0.986; RMSEA=0.095, 90% CI 0.033-0.176; *P*=.11; SRMR=0.039; Δχ^2^_3_=35.7; *P*<.001; anxiety symptoms: χ^2^_1_=6.0; *P*=.01; CFI=0.985; RMSEA=0.097, 90% CI 0.035-0.178; *P*=.10; SRMR=0.040; Δχ^2^_3_=30.0; *P*<.001), supporting differences in LCSs among the groups. The LCS estimates (Table 2) suggest a statistically significant decrease in depressive symptoms from baseline to after the intervention in both groups. However, the decrease is 2.9-fold higher in the intervention group. Moreover, a statistically significant decrease in anxiety symptoms from T0 to T1 was found only in the intervention group. Changes were found to be heterogeneous across individuals in both the groups (Table 2). Furthermore, the covariance between the levels of anxiety and depressive symptoms at T0 and the amount of change over time were statistically significant (*P*<.001; Table 2), suggesting that the higher the depressive and anxiety symptoms at baseline, the greater the decrease in change scores. Subgroup analyses were performed considering 2 subgroups: evaluative respondents (women who completed both assessments) and women presenting an EPDS score of >9 at baseline. Concerning the evaluative respondents, the unconstrained model presented a better fit to the data than the constrained model for both depressive (Δχ^2^_3_=179.0; *P*<.001) and anxiety symptoms (Δχ^2^_3_=35.3; *P*<.001). The women in the intervention group reported a statistically significant higher decrease in depressive (intervention group: µΔ=–2.41; *P*<.001 vs control group: µΔ=–0.71; *P*<.001) and anxiety symptoms (intervention group: µΔ=–2.41; *P*<.001 vs control group: µΔ=–0.71; *P*=.74). Concerning the women presenting an EPDS score of >9 at baseline, the unconstrained model also presented a better fit to the data than the constrained model for depressive (Δχ^2^_3_=88.9; *P*<.001) and anxiety symptoms (Δχ^2^_3_=27.8; *P*<.001). Once again, the women in the intervention group reported a significantly higher decrease in depressive (intervention group: µΔ=–3.35; *P*<.001 vs control group: µΔ=–1.48; *P*<.001) and anxiety symptoms (intervention group: µΔ=–2.24; *P*<.001 vs control group: µΔ=–0.43; *P*=.04).

Concerning psychological processes (emotion regulation, self-compassion, and psychological flexibility) too, the unconstrained models presented a better model fit than the full constrained models, suggesting differences in LCS estimates among the groups. Specifically, for emotion regulation (full constrained model: χ^2^_4_=18.0; *P*<.001; CFI=0.965; RMSEA=0.082, 90% CI 0.046-0.122; *P*<.001; SRMR=0.072; unconstrained model: χ^2^_1_=5.7; *P*=.02; CFI=0.988; RMSEA=0.095, 90% CI 0.03-0.176; *P*=.11; SRMR=0.041; Δχ^2^_3_=12.3; *P*=.007), the LCSs suggest a statistically significant decrease in emotion regulation difficulties from T0 to T1 in the intervention group but not in the control group. Concerning self-compassion (full constrained model: χ^2^_4_=68.1; *P*<.001; CFI=0.889; RMSEA=0.174, 90% CI 0.140-0.212; *P*<.001; SRMR=0.160; unconstrained model: χ^2^_1_=2.6; *P*=.11; CFI=0.997; RMSEA=0.054, 90% CI 0.000-0.141; *P*=.33; SRMR=0.027; Δχ^2^_3_=65.6; *P*<.001), the LCS estimates suggest a statistically significant increase in the levels of self-compassion over time in both groups. However, the increase is 4.5-fold higher in the intervention group. Finally, concerning psychological flexibility (full constrained model: χ^2^_4_=41.1; *P*<.001; CFI=0.923; RMSEA=0.133, 90% CI 0.098-0.171; *P*<.001; SRMR=0.05; unconstrained model: χ^2^_1_=6.2; *P*=.01; CFI=0.989; RMSEA=0.099, 90% CI 0.360-0.180; *P*=.09; SRMR=0.041; Δχ^2^_3_=34.9; *P*<.001), a statistically significant increase in the levels of psychological flexibility over time was found in the intervention group but not in the control group (Table 2). Changes were found to be heterogeneous across individuals in both groups. Furthermore, the covariance between the levels of emotion regulation, self-compassion, and psychological flexibility at T0 and amount of change over time were statistically significant (Table 2), suggesting that the higher the emotion regulation difficulties at baseline, the greater the decrease in emotion regulation difficulties over time, and the lower the self-compassion and psychological flexibility at baseline, the greater the increase in these variables over time. Subgroup analyses were also performed. Concerning the evaluative respondents, the unconstrained models presented a better fit to the data than the constrained models for all the variables (emotion regulation: Δχ^2^_3_=8.5; *P*<.001; self-compassion: Δχ^2^_3_=59.4; *P*<.001; and psychological flexibility: Δχ^2^_3_=32.3; *P*<.001). The LCS estimates were different across the groups: in the intervention group, women reported a statistically significant decrease in emotion regulation difficulties (µΔ=–1.506; *P*=.047) and a statistically significant increase in self-compassion (µΔ=2.585; *P*<.001) and psychological flexibility (µΔ=2.490; *P*=.003), whereas no statistically significant changes were found in the control group (emotion regulation: µΔ=–0.689; *P*=.25; self-compassion: µΔ=0.537; *P*=.06; and psychological flexibility: µΔ=0.474; *P*=.32). Concerning the women presenting an EPDS score of >9 at baseline, a similar pattern was found with the unconstrained models presenting a better fit to the data for all the variables than the constrained models (emotion regulation: Δχ^2^_3_=10.9; *P*<.001; self-compassion: Δχ^2^_3_=44.4; *P*<.001; and psychological flexibility: Δχ^2^_3_=25.4; *P*<.001). In addition, the women in the intervention group reported a statistically significant decrease in emotion regulation difficulties (µΔ=–3.748; *P*<.001) and a statistically significant increase in self-compassion (µΔ=3.229; *P*<.001) and psychological flexibility (µΔ=4.880; *P*<.001), whereas the women in the control group reported no statistically significant change in emotion regulation (µΔ=–0.264; *P*=.70) or self-compassion (µΔ=0.499; *P*=.16) and a statistically significant but smaller change only in psychological flexibility (µΔ=1.158; *P*=.049). In fact, when we analyze the LCS estimates of the total sample and of the women presenting an EPDS score of >9 at baseline, we observe a higher change in psychological processes among the women presenting clinically relevant depressive symptoms who participate in the *Be a Mom* intervention. See Multimedia Appendix 2, Table S1, for exact *P* values.

**Table 2:** Changes in adjustment outcomes and psychological processes over time in the intervention and control groups: descriptive statistics and univariate latent change score (LCS) estimates.

	Descriptive statistics				Univariate LCS estimates^a^					
	Intervention group, mean (SD)		Control group, mean (SD)		Intervention group, B (SE)			Control group, B (SE)		
	T0^b^ (n=542)	T1^c^ (n=267)	T0 (n*=*511*)*	T1 (n=445)	μ_Δ_^d^	σ^2^_Δ_^e^	σ1	μ_Δ_	σ^2^_Δ_	σ1
EPDS^f^	10.99 (5.14)	8.75 (4.53)	11.73 (4.81)	10.78 (5.06)	–2.27 (0.24)***	20.11 (1.71)***	–12.97 (1.43)***	–0.79 (0.19)***	16.67 (1.12)***	–6.92 (0.99)***
HADS-A^g^	7.77 (4.13)	6.57 (3.93)	8.40 (4.21)	8.24 (4.21)	–1.36 (0.20)***	13.53 (1.16)***	–7.53 (0.95)***	0.00 (0.16)	12.61 (0.85)***	–.62 (0.77)***
DERS^h^	42.28 (13.29)	39.47 (13.07)	44.26 (13.50)	42.77 (13.87)	–2.87 (0.71)***	135.62 (12.59)***	–69.88 (10.26)***	–0.82 (0.48)	103.71 (7.22)***	–44.13 (7.15)***
SCS-SF^i^	35.80 (8.95)	34.93 (8.64)	38.17 (8.68)	35.65 (9.41)	2.90 (0.46)***	57.25 (5.21)***	–32.77 (4.25)***	0.65 (0.27)*	29.35 (2.06)***	–8.96 (2.31)***
CompACT^j^	58.56 (14.33)	61.30 (15.53)	56.36 (14.47)	57.25 (15.11)	2.82 (0.83)**	185.18 (16.58)***	–79.15 (12.04)***	0.37 (0.48)	103.41 (7.07)***	–45.51 (7.26)***

^a^The latent change score estimates presented correspond to the unconstrained model (where the latent change score parameters were free to vary across the groups, except for means at baseline, which were constrained to be equal across the groups). Unstandardized estimates are presented.

^b^T0: baseline assessment.

^c^T1: postintervention assessment.

^d^μ_Δ_: mean intercept of the latent change factor.

^e^σ^2^_Δ_: variance of the latent change factor.

^f^EPDS: Edinburgh Postpartum Depression Scale (depressive symptoms).

^g^HADS-A: Hospital Anxiety and Depression Scale, anxiety subscale (anxiety symptoms).

^h^DERS: Difficulties in Emotion Regulation Scale (emotion regulation difficulties).

^i^SCS-SF: Self-Compassion Scale-Short Form (self-compassion).

^j^CompACT: Comprehensive Assessment of Acceptance and Commitment Therapy Processes (psychological flexibility).

*P<.05.

**P<.01.

***P<.001.

### Change-to-Change Effects of Psychological Processes on Adjustment Outcomes

A 2W-LCS model was tested, in which we computed not only the univariate LCS for each variable but also the change-to-change effects of the psychological processes (emotion regulation, self-compassion, and psychological flexibility) on the adjustment outcomes (depressive and anxiety symptoms). A multigroup-model approach was used to test whether the path models were similar or different across the intervention and control groups. The first model to be tested was the fully constrained model (model A, in which the univariate LCSs, the cross-lagged effects, and the change-to-change effects were constrained to be equal across the groups). This model presented an acceptable fit to the data (χ^2^_103_=326.3; *P*<.001; CFI=0.946; RMSEA=0.064, 90% CI 0.056-0.072; *P*=.002; SRMR=0.078). The second model to be tested (model B) was the model in which the LCSs and the cross-lagged effects were constrained to be equal across the groups, but the change-to-change effects were free to vary across the groups. This model presented a slightly better fit to the data than the fully constrained model (Δχ^2^_4_=3.7; *P*<.001; χ^2^_99_=322.7; *P*<.001; CFI=0.946; RMSEA=0.066, 90% CI 0.058-0.074; *P*=.001; SRMR=0.078), suggesting that change-to-change effects differ across the groups. The third model to be tested (model C) was the model in which the LCSs were constrained to be equal across the groups, but the cross-lagged effects and the change-to-change effects were free to vary across the groups. Compared with model B, model C presented a better fit to the data (Δχ^2^_12_=21.2; *P*<.001; χ^2^_87_=301.5; *P*<.001; CFI=0.948; RMSEA=0.068, 90% CI 0.060-0.077; *P*=.001; SRMR=0.076). Finally, the unconstrained model (model D, in which the LCSs, cross-lagged effects, and change-to-change effects were free to vary across the groups) was tested and compared with model C, presenting a statistically significantly better fit to the data (Δχ^2^_5_=48.6; *P*<.001; χ^2^_82_=252.8; *P*<.001; CFI=0.96; RMSEA=0.063, 90% CI 0.054-0.072; *P*=.001; SRMR=0.072), which suggests that the univariate LCSs, the cross-lagged effects, and the change-to-change effects were different across the groups. Table 3 presents the standardized estimates of the different parameters for both the intervention and control groups.

Concerning correlations among the variables at baseline, statistically significant and large associations were found in both groups (Table 3). Of note, higher levels of emotion regulation difficulties and lower levels of self-compassion and psychological flexibility were statistically significantly associated with higher levels of depressive and anxiety symptoms at baseline in both groups.

Concerning cross-lagged effects, different patterns were found in the intervention and control groups. In the intervention group, higher levels of emotion regulation difficulties at baseline were associated with a higher change in depressive and anxiety symptoms, and higher levels of self-compassion and psychological flexibility at baseline were associated with a lower change in anxiety symptoms. Moreover, higher levels of depressive symptoms at baseline were associated with a significantly higher change in the levels of self-compassion and psychological flexibility. Conversely, in the control group, higher levels of emotion regulation difficulties at baseline were associated with a higher change in anxiety symptoms, and higher levels of psychological flexibility at baseline were associated with a higher change in depressive and anxiety symptoms. In addition, in the control group, higher levels of anxiety and depressive symptoms were associated with a lower change in the levels of emotion regulation over time (Table 3).

Concerning correlations among the change effects (univariate LCSs), statistically significant and moderate associations were found between changes in depressive symptoms and changes in anxiety symptoms in both groups (intervention group: B=0.377, SE 0.053; *P*<.001; control group: B=0.356, SE 0.042; *P*<.001), suggesting that the higher the decrease in depressive symptoms, the higher the decrease in anxiety symptoms. Moreover, statistically significant associations were found among changes in psychological processes in both groups, with a higher decrease in emotion regulation difficulties being associated with a higher increase in self-compassion and psychological flexibility and a higher increase in self-compassion being associated with a higher increase in psychological flexibility. However, the strength of the associations seems to be higher in the intervention group than in the control group (Table 3).

Finally, concerning change-to-change effects, a higher increase in self-compassion and psychological flexibility was statistically significantly associated with a higher reduction in depressive and anxiety symptoms in both the intervention and control groups. Moreover, a higher decrease in emotion regulation difficulties was associated with a higher reduction in anxiety symptoms in the intervention group and with a higher reduction in both depressive and anxiety symptoms in the control group (Table 3).

**Table 3:** Two-wave latent change score–model estimates: correlations at baseline, cross-lagged effects, and change-to-change effects in the intervention group and control group.

		Intervention group		Control group	
		B (SE)	*P* value	B (SE)	*P* value
**Correlations between variables at baseline**					
	EPDS^a^_T0_^b^ ↔HADS-A^c^_T0_	0.721 (0.021)	<.001	0.711 (0.022)	<.001
	EPDS_T0_ ↔ DERS^d^_T0_	0.609 (0.026)	<.001	0.610 (0.027)	<.001
	EPDS_T0_ ↔ SCS-SF^e^_T0_	–0.566 (0.028)	<.001	–0.565 (0.030)	<.001
	EPDS_T0_ ↔ CompACT^f^_T0_	–0.594 (0.027)	<.001	–0.580 (0.029)	<.001
	HADS-A_T0_ ↔ DERS_T0_	0.604 (0.027)	<.001	0.508 (0.033)	<.001
	HADS-A_T0_ ↔ SCS-SF_T0_	–0.562 (0.030)	<.001	–0.506 (0.033)	<.001
	HADS-A_T0_ ↔ CompACT_T0_	–0.574 (0.029)	<.001	–0.535 (0.031)	<.001
	DERS_T0_ ↔ SCS-SF_T0_	–0.720 (0.021)	<.001	–0.714 (0.022)	<.001
	DERS_T0_ ↔ CompACT_T0_	–0.651 (0.025)	<.001	–0.661 (0.025)	<.001
	SCS-SF_T0_ ↔ CompACT_T0_	0.589 (0.028)	<.001	0.638 (0.027)	<.001
**Cross-lagged effects between psychological processes at T0 and change in adjustment outcomes**					
	DERS_T0_ → EPDS change^g^	0.087 (0.043)	.04	0.038 (0.040)	.34
	SCS-SF_T0_ → EPDS change	–0.042 (0.043)	.33	–0.061 (0.040)	.13
	CompACT_T0_ → EPDS change	–0.059 (0.044)	.18	–0.147 (0.038)	<.001
	DERS_T0_ → HADS-A change^h^	0.213 (0.037)	<.001	0.121 (0.034)	<.001
	SCS-SF_T0_ → HADS-A change	–0.162 (0.040)	<.001	–0.058 (0.037)	.12
	CompACT_T0_ → HADS-A change	–0.089 (0.041)	.03	–0.115 (0.035)	.001
**Cross-lagged effects between adjustment outcomes at T0 and change in psychological processes**					
	EPDS_T0_ → DERS change^i^	–0.064 (0.051)	.21	–0.115 (0.041)	.005
	HADS-A_T0_ → DERS change	–0.098 (0.054)	.07	–0.105 (0.045)	.02
	EPDS_T0_ → SCS-SF change^j^	0.101 (0.051)	.046	0.009 (0.043)	.84
	HADS-A_T0_ → SCS-SF change	0.057 (0.054)	.29	–0.002 (0.047)	.96
	EPDS_T0_ → CompACT change^k^	0.114 (0.051)	.02	0.056 (0.041)	.17
	HADS-A_T0_ → CompACT change	0.073 (0.054)	.17	0.061 (0.044)	.17
**Correlations between changes in psychological processes**					
	DERS change ↔ SCS-SF change	–0.556 (0.044)	<001	–0.510 (0.037)	<.001
	DERS change ↔ CompACT change	–0.490 (0.049)	<.001	–0.362 (0.043)	<.001
	SCS-SF change ↔ CompACT change	0.492 (0.049)	<.001	0.303 (0.045)	<.001
**Change-to-change effects**					
	DERS change → EPDS change	0.099 (0.065)	.13	0.255 (0.050)	<.001
	SCS-SF change → EPDS change	–0.245 (0.065)	<.001	–0.101 (0.050)	.045
	CompACT change → EPDS change	–0.333 (0.060)	<.001	–0.276 (0.044)	<.001
	DERS change → HADS-A change	0.219 (0.062)	.001	0.198 (0.053)	<.001
	SCS-SF change → HADS-A change	–0.172 (0.063)	.006	–0.107 (0.053)	.04
	CompACT change → HADS-A change	–0.290 (–0.059)	<.001	–0.262 (0.046)	<.001

^a^EPDS: Edinburgh Postpartum Depression Scale (depressive symptoms).

^b^T0: baseline.

^c^HADS*-*A: Hospital Anxiety and Depression Scale, anxiety subscale (anxiety symptoms).

^d^DERS: Difficulties in Emotion Regulation Scale (emotion regulation difficulties).

^e^SCS-SF: Self-Compassion Scale-Short Form (self-compassion).

^f^CompACT: Comprehensive Assessment of Acceptance and Commitment Therapy Processes (psychological flexibility).

^g^EPDS change: change in depressive symptoms over time.

^h^HADS-A change: change in anxiety symptoms over time.

^i^DERS change: change in emotion regulation difficulties over time.

^j^SCS-SF change: change in self-compassion over time.

^k^CompACT change: change in psychological flexibility over time.

## Discussion

### Principal Findings

This trial investigated the mechanisms of change associated with the efficacy of *Be a Mom*, a web-based preventive intervention, in postpartum women at high risk for PPD. *Be a Mom* was found to be uniquely effective in reducing depressive and anxiety symptoms from baseline to after the intervention in comparison with the control group where such changes were inexistent or much smaller. This pattern of symptom reduction was similar when considering only the participants who completed both assessments and when considering women presenting clinically relevant symptoms of depression (ie, EPDS score of >9) at baseline. This finding consolidates and extends previous evidence for the efficacy of *Be a Mom* in targeting both clusters of symptoms [14]. Moreover, the fact that higher levels of psychopathological symptoms at baseline were related to greater posttreatment improvements suggests that this low-intensity program may be a promising first-line intervention for helping women considered to be at risk, particularly those with early-onset PPD symptoms.

In addition to the positive effects that *Be a Mom* revealed at the level of symptom reduction, the examination of concomitant psychological processes was conducted as a crucial step in producing a reliable account of the mechanisms involved in the change processes [12]. All the 3 psychological processes under study improved statistically significantly (with greater enhancements over time related to greater impairments at the baseline) in posttreatment assessments: emotion regulation ability and psychological flexibility improved only in the intervention group, and although self-compassion increased in both groups, these improvements were considerably greater in the intervention group. These psychological processes were nuclear processes that were expected to be promoted through *third wave* CBT approaches [13] and therefore in line with the therapeutic framework that supported the development of the *Be a Mom* program. Moreover, a higher increase in self-compassion and psychological flexibility was associated with greater overall symptom reduction, although a higher decrease in emotion regulation difficulties was related to a reduction in anxiety symptoms only. This pattern of associations is consistent with previous reports of difficulties in emotion regulation (as assessed herein) being distinctively related to anxiety symptoms, even when depressive symptoms are controlled [35].

In a systematic review of CBT interventions to prevent PPD, Werner et al [16] noted that 4 out of 6 studies reporting success in the alleviation of PPD targeted only women considered to be at risk (with sample sizes ranging from 27 to 241 participants). Nevertheless, none of these face-to-face studies examined the mechanisms of change underlying the observed reductions in depressive symptoms. The results from our study were based on a large sample of women at high risk for PPD and make important contributions to the field by ascertaining the links between enhanced self-regulatory skills and improved perinatal mental health outcomes in a web-based CBT individual intervention that was specifically devised to prevent PPD. It bears recalling that most web-based interventions for PPD were not specifically designed or tested for preventive purposes and that they relied exclusively on classic CBT [7,10,11]; moreover, 2 of the few internet-based CBT interventions for the prevention of PPD—*Mothers and Babies Course* and *Sunnyside*—also relied exclusively on traditional CBT, with the former resulting from an adaptation of a group-based face-to-face intervention [8] and the latter resulting from an adaptation of a group-based internet intervention [9]. In both cases, the programs encompassed 8 sessions, and the examination of the effects of the preventive interventions revealed that they failed to reach statistical significance in the respective RCTs [8,9].

Currently, the available internet-based interventions for PPD are mostly based on CBT, and even if the effect of CBT alone cannot be fully ascertained regarding the prevention of PPD, the overall trend gives cause for optimism, provided that the development of such programs is tailored to the needs and expectations of end users [36]. Taken together, this study contributes to the existing literature on CBT interventions to prevent PPD in 3 ways: first, it gathered compelling evidence for the efficacy of *Be a Mom* in reducing depressive and anxiety symptoms in a fairly large sample of women identified as being at high risk for PPD; second, it determined the role of self-regulatory psychological processes in the putative mechanisms of change linking participation in the CBT-based *Be a Mom* intervention with improved mental health outcomes; and third, it lends support to a preventive web-based intervention that was originally developed with the integration of classic and *third wave* CBT elements and methodically based on end users’ appraisals [14,37].

### Limitations

This RCT provided good evidence for the efficacy of *Be a Mom* and related mechanisms of change, from which causal and functional accounts can be further developed. Nevertheless, a few limitations should be considered in this study. First, web-based recruitment was the preferred method for enrolling participants at the cost of excluding women with the lowest levels of digital literacy as well as those who, through lack of interest or opportunity, do not use social media at all. The recruitment strategy targeted women through specific advertisements, thus being prone to volunteer bias. Moreover, although the women were instructed to seek no other intervention besides *Be a Mom* or usual care while participating in the RCT, the access to other web-based resources was not monitored. Second, eligible women were assessed on the web regarding their risk for developing PPD, which may also increase the risk for self-selection bias (ie, women with greater interest in the study or experiencing higher distress may have been prone to participate, thus not ensuring the representativity of all women at risk for PPD in the resultant sample). Third, the dropout rate after the treatment (341/1053, 32.23%, in the total sample) was higher in the intervention group than in the control group. This could be because no compensation was offered in the study, and most women in the intervention group who dropped out from the *Be a Mom* program reported lack of time as the main reason for dropping out (suggesting that they may not have had the time to complete the assessment protocol). Despite this imbalance, both groups presented similar sociodemographic characteristics even when considering only women who completed both assessments, and the characteristics that distinguish completers and dropouts (in the total sample) were introduced as covariates in the analyses. Fourth and last, data were exclusively collected through self-report measures, which may be easily affected by social desirability factors (eg, the myths of *perfect motherhood*).

### Conclusions and Future Directions

This study demonstrated the efficacy of *Be a Mom* in reducing the psychological maladjustment of postpartum women considered to be at risk for PPD through the enhancement of core psychological processes such as emotion regulation, psychological flexibility, and self-compassion. To provide further evidence of the efficacy of *Be a Mom* in preventing PPD, more follow-up assessments are needed (up to 12 months post partum), while controlling for the time between assessments in the respective analyses. According to the criteria suggested by the American Psychological Association (Division 12 Task Force on Psychological Interventions) for empirically validated treatments, the conduction of additional RCTs would be desirable to eventually ascertain *Be a Mom* as a well-established intervention. Moreover, *Be a Mom* was recently investigated as a universal preventive intervention, with, as in our study, promising results in the promotion of positive mental health among postpartum women with low risk for PPD [38].

## Appendix

Multimedia Appendix 1 CONSORT-eHEALTH checklist (V 1.6.1).

Multimedia Appendix 2 Table S1. Changes in adjustment outcomes and psychological processes over time in the intervention and control groups: descriptive statistics and univariate latent change score (LCS) estimates.

## Abbreviations

2W-LCS: 2-wave latent change score
CBT: cognitive behavioral therapy
CFI: comparative fit index
CompACT: Comprehensive Assessment of Acceptance and Commitment Therapy Processes
CONSORT-eHEALTH: Consolidated Standards of Reporting Trials of Electronic and Mobile Health Applications and Online Telehealth
EPDS: Edinburgh Postpartum Depression Scale
LCS: latent change score
PDPI-R: Postpartum Depression Predictors Inventory-Revised
PPD: postpartum depression
RCT: randomized controlled trial
RMSEA: root mean square of approximation
SCS-SF: Self-Compassion Scale-Short Form
SRMR: standardized root mean square residual

## Data Availability

The data that support the findings of this study are available from the corresponding author upon reasonable request.
