# Supplementary material for: The Efficacy of Be a Mom, a Web-Based Intervention to Prevent Postpartum Depression: Examining Mechanisms of Change in a Randomized Controlled Trial
Source: JMIR Ment Health. 2023 Mar 17;10:e39253. doi: 10.2196/39253 (PMC10139682; doi:10.2196/39253)
Supplement: Multimedia Appendix 1 [file mental_v10i1e39253_app1.pdf]

CONSORT-EHEALTH (V 1.6.1) - Submission/Publication Form

The CONSORT-EHEALTH checklist is intended for authors of randomized trials evaluating web-based and Internet-based applications/interventions, including mobile interventions, electronic games (incl multiplayer games), social media, certain telehealth applications, and other interactive and/or networked electronic applications. Some of the items (e.g. all subitems under item 5 - description of the intervention) may also be applicable for other study designs.

The goal of the CONSORT EHEALTH checklist and guideline is to be
a) a guide for reporting for authors of RCTs,
b) to form a basis for appraisal of an ehealth trial (in terms of validity)

CONSORT-EHEALTH items/subitems are MANDATORY reporting items for studies published in the Journal of Medical Internet Research and other journals / scientific societies endorsing the checklist.

Items numbered 1., 2., 3., 4a., 4b etc are original CONSORT or CONSORT-NPT (non-pharmacologic treatment) items.
Items with Roman numerals (i., ii, iii, iv etc.) are CONSORT-EHEALTH extensions/clarifications.

As the CONSORT-EHEALTH checklist is still considered in a formative stage, we would ask that you also RATE ON A SCALE OF 1-5 how important/useful you feel each item is FOR THE PURPOSE OF THE CHECKLIST and reporting guideline (optional).

Mandatory reporting items are marked with a red *.
In the textboxes, either copy & paste the relevant sections from your manuscript into this form - please include any quotes from your manuscript in QUOTATION MARKS,
or answer directly by providing additional information not in the manuscript, or elaborating on why the item was not relevant for this study.

YOUR ANSWERS WILL BE PUBLISHED AS A SUPPLEMENTARY FILE TO YOUR PUBLICATION IN JMIR AND ARE CONSIDERED PART OF YOUR PUBLICATION (IF ACCEPTED).
Please fill in these questions diligently. Information will not be copyedited, so please use proper spelling and grammar, use correct capitalization, and avoid abbreviations.

DO NOT FORGET TO SAVE AS PDF _AND_ CLICK THE SUBMIT BUTTON SO YOUR ANSWERS ARE IN OUR DATABASE !!!

Citation Suggestion (if you append the pdf as Appendix we suggest to cite this paper in the caption):
Eysenbach G, CONSORT-EHEALTH Group
CONSORT-EHEALTH: Improving and Standardizing Evaluation Reports of Web-based and Mobile Health Interventions
J Med Internet Res 2011;13(4):e126

URL: http://www.jmir.org/2011/4/e126/
doi: 10.2196/jmir.1923
PMID: 22209829

carona.carlos@gmail.com (não partilhado) Mudar de conta

Rascunho guardado

* Obrigatório

Your name *

First Last

Carlos Carona

Primary Affiliation (short), City, Country *

University of Toronto, Toronto, Canada

University of Coimbra

Your e-mail address *

abc@gmail.com

carona.carlos@gmail.com

Title of your manuscript *

Provide the (draft) title of your manuscript.

The Efficacy of “Be a Mom”, a Web-based Intervention to Prevent Postpartum Depression: Examining Mechanisms of Change in a Randomized Controlled Trial

Name of your App/Software/Intervention *

If there is a short and a long/alternate name, write the short name first and add the long name in brackets.

"Be a Mom" (Web-based Intervention to Prever

Evaluated Version (if any)

e.g. "V1", "Release 2017-03-01", "Version 2.0.27913"

Original version

Language(s) *

What language is the intervention/app in? If multiple languages are available, separate by comma (e.g. "English, French")

Portuguese; English

URL of your Intervention Website or App

e.g. a direct link to the mobile app on app in appstore (itunes, Google Play), or URL of the website. If the intervention is a DVD or hardware, you can also link to an Amazon page.

https://beamom.pt/

URL of an image/screenshot (optional)

https://pt-pt.facebook.com/beamom.pt/

Accessibility *

Can an enduser access the intervention presently?

- access is free and open
- access only for special usergroups, not open
- access is open to everyone, but requires payment/subscription/in-app purchases
- app/intervention no longer accessible
- Outra:

Primary Medical Indication/Disease/Condition *

e.g. "Stress", "Diabetes", or define the target group in brackets after the condition, e.g. "Autism (Parents of children with)", "Alzheimers (Informal Caregivers of)"

Postpartum Depression

Primary Outcomes measured in trial *

comma-separated list of primary outcomes reported in the trial

Depressive symptoms, anxiety symptoms

Secondary/other outcomes

Are there any other outcomes the intervention is expected to affect?

Emotion regulation skills, psychological flexibility, self-compassion

Recommended "Dose" *

What do the instructions for users say on how often the app should be used?

- ( ) Approximately Daily
- (x) Approximately Weekly
- ( ) Approximately Monthly
- ( ) Approximately Yearly
- ( ) "as needed"
- ( ) Outra:

Approx. Percentage of Users (starters) still using the app as recommended after 3 months *

- (x) unknown / not evaluated
- ( ) 0-10%
- ( ) 11-20%
- ( ) 21-30%
- ( ) 31-40%
- ( ) 41-50%
- ( ) 51-60%
- ( ) 61-70%
- ( ) 71%-80%
- ( ) 81-90%
- ( ) 91-100%
- ( ) Outra:

Overall, was the app/intervention effective? *

- (x) yes: all primary outcomes were significantly better in intervention group vs control
- ( ) partly: SOME primary outcomes were significantly better in intervention group vs control
- ( ) no statistically significant difference between control and intervention
- ( ) potentially harmful: control was significantly better than intervention in one or more outcomes
- ( ) inconclusive: more research is needed
- ( ) Outra:

Article Preparation Status/Stage *

At which stage in your article preparation are you currently (at the time you fill in this form)

- ( ) not submitted yet - in early draft status
- ( ) not submitted yet - in late draft status, just before submission
- ( ) submitted to a journal but not reviewed yet
- ( ) submitted to a journal and after receiving initial reviewer comments
- (x) submitted to a journal and accepted, but not published yet
- ( ) published
- ( ) Outra:

Journal *

If you already know where you will submit this paper (or if it is already submitted), please provide the journal name (if it is not JMIR, provide the journal name under "other")

- ( ) not submitted yet / unclear where I will submit this
- ( ) Journal of Medical Internet Research (JMIR)
- ( ) JMIR mHealth and UHealth
- ( ) JMIR Serious Games
- (x) JMIR Mental Health
- ( ) JMIR Public Health
- ( ) JMIR Formative Research
- ( ) Other JMIR sister journal
- ( ) Outra:

Is this a full powered effectiveness trial or a pilot/feasibility trial? *

- ( ) Pilot/feasibility
- (x) Fully powered

Manuscript tracking number *

If this is a JMIR submission, please provide the manuscript tracking number under "other" (The ms tracking number can be found in the submission acknowledgement email, or when you login as author in JMIR. If the paper is already published in JMIR, then the ms tracking number is the four-digit number at the end of the DOI, to be found at the bottom of each published article in JMIR)

- ( ) no ms number (yet) / not (yet) submitted to / published in JMIR
- (x) Outra: 39253

TITLE AND ABSTRACT

1a) TITLE: Identification as a randomized trial in the title

1a) Does your paper address CONSORT item 1a? *

I.e does the title contain the phrase "Randomized Controlled Trial"? (if not, explain the reason under "other")

- (x) yes
- ( ) Outra:

1a-i) Identify the mode of delivery in the title

Identify the mode of delivery. Preferably use “web-based” and/or “mobile” and/or “electronic game” in the title. Avoid ambiguous terms like “online”, “virtual”, “interactive”. Use “Internet-based” only if Intervention includes non-web-based Internet components (e.g. email), use “computer-based” or “electronic” only if offline products are used. Use “virtual” only in the context of “virtual reality” (3-D worlds). Use “online” only in the context of “online support groups”. Complement or substitute product names with broader terms for the class of products (such as “mobile” or “smart phone” instead of “iphone”), especially if the application runs on different platforms.

subitem not at all important

- 1 ( )
- 2 ( )
- 3 ( )
- 4 ( )
- 5 (x)

essential

Limpar seleção

Does your paper address subitem 1a-i? *

Copy and paste relevant sections from manuscript title (include quotes in quotation marks "like this" to indicate direct quotes from your manuscript), or elaborate on this item by providing additional information not in the ms, or briefly explain why the item is not applicable/relevant for your study

"The Efficacy of “Be a Mom”, a Web-based Intervention to Prevent Postpartum Depression: Examining Mechanisms of Change in a Randomized Controlled Trial"

1a-ii) Non-web-based components or important co-interventions in title

Mention non-web-based components or important co-interventions in title, if any (e.g., “with telephone support”).

subitem not at all important

- 1 ○
- 2 ○
- 3 ◉
- 4 ○
- 5 ○

essential

Limpar seleção

Does your paper address subitem 1a-ii?

Copy and paste relevant sections from manuscript title (include quotes in quotation marks "like this" to indicate direct quotes from your manuscript), or elaborate on this item by providing additional information not in the ms, or briefly explain why the item is not applicable/relevant for your study

Not applicable; there were no additional interventions to the web-based program.

1a-iii) Primary condition or target group in the title

Mention primary condition or target group in the title, if any (e.g., “for children with Type I Diabetes”) Example: A Web-based and Mobile Intervention with Telephone Support for Children with Type I Diabetes: Randomized Controlled Trial

subitem not at all important

1 ○

2 ○

3 ○

4 ○

5 ◉

essential

Limpar seleção

Does your paper address subitem 1a-iii? *

Copy and paste relevant sections from manuscript title (include quotes in quotation marks "like this" to indicate direct quotes from your manuscript), or elaborate on this item by providing additional information not in the ms, or briefly explain why the item is not applicable/relevant for your study

"The Efficacy of “Be a Mom”, a Web-based Intervention to Prevent Postpartum Depression: Examining Mechanisms of Change in a Randomized Controlled Trial"

1b) ABSTRACT: Structured summary of trial design, methods, results, and conclusions

NPT extension: Description of experimental treatment, comparator, care providers, centers, and blinding status.

1b-i) Key features/functionalities/components of the intervention and comparator in the METHODS section of the ABSTRACT

Mention key features/functionalities/components of the intervention and comparator in the abstract. If possible, also mention theories and principles used for designing the site. Keep in mind the needs of systematic reviewers and indexers by including important synonyms. (Note: Only report in the abstract what the main paper is reporting. If this information is missing from the main body of text, consider adding it)

subitem not at all important

1 ○

2 ○

3 ○

4 ○

5 ◉

essential

Limpar seleção

Does your paper address subitem 1b-i? *

Copy and paste relevant sections from the manuscript abstract (include quotes in quotation marks "like this" to indicate direct quotes from your manuscript), or elaborate on this item by providing additional information not in the ms, or briefly explain why the item is not applicable/relevant for your study

"This two-arm, open-label randomized controlled trial (RCT) involved a sample of 1058 perinatal women presenting high-risk for PPD, who were allocated to “Be a Mom” intervention group or a waiting-list control group, and completed self-report measures at baseline and post-intervention assessments. Univariate Latent Change Score (LCS) models were computed to determine changes over time in adjustment processes and outcomes, with a multigroup model approach to detect differences between the intervention and the groups, and a two-wave LCS model to examine whether changes in processes were related to changes in outcomes."

1b-ii) Level of human involvement in the METHODS section of the ABSTRACT

Clarify the level of human involvement in the abstract, e.g., use phrases like “fully automated” vs. “therapist/nurse/care provider/physician-assisted” (mention number and expertise of providers involved, if any). (Note: Only report in the abstract what the main paper is reporting. If this information is missing from the main body of text, consider adding it)

subitem not at all important

1 ○

2 ○

3 ○

4 ○

5 ◉

essential

Limpar seleção

Does your paper address subitem 1b-ii?

Copy and paste relevant sections from the manuscript abstract (include quotes in quotation marks "like this" to indicate direct quotes from your manuscript), or elaborate on this item by providing additional information not in the ms, or briefly explain why the item is not applicable/relevant for your study

"This two-arm, open-label randomized controlled trial (RCT) involved a sample of 1058 perinatal women presenting high-risk for PPD, who were allocated to “Be a Mom” intervention group or a waiting-list control group, and completed self-report measures at baseline and post-intervention assessments."

1b-iii) Open vs. closed, web-based (self-assessment) vs. face-to-face assessments in the METHODS section of the ABSTRACT

Mention how participants were recruited (online vs. offline), e.g., from an open access website or from a clinic or a closed online user group (closed usergroup trial), and clarify if this was a purely web-based trial, or there were face-to-face components (as part of the intervention or for assessment). Clearly say if outcomes were self-assessed through questionnaires (as common in web-based trials). Note: In traditional offline trials, an open trial (open-label trial) is a type of clinical trial in which both the researchers and participants know which treatment is being administered. To avoid confusion, use “blinded” or “unblinded” to indicated the level of blinding instead of “open”, as “open” in web-based trials usually refers to “open access” (i.e. participants can self-enrol). (Note: Only report in the abstract what the main paper is reporting. If this information is missing from the main body of text, consider adding it)

subitem not at all important

1 ○

2 ○

3 ○

4 ◉

5 ○

essential

Limpar seleção

Does your paper address subitem 1b-iii?

Copy and paste relevant sections from the manuscript abstract (include quotes in quotation marks "like this" to indicate direct quotes from your manuscript), or elaborate on this item by providing additional information not in the ms, or briefly explain why the item is not applicable/relevant for your study

"This two-arm, open-label randomized controlled trial (RCT) involved a sample of 1058 perinatal women presenting high-risk for PPD, who were allocated to “Be a Mom” intervention group or a waiting-list control group, and completed self-report measures at baseline and post-intervention assessments."

1b-iv) RESULTS section in abstract must contain use data

Report number of participants enrolled/assessed in each group, the use/uptake of the intervention (e.g., attrition/adherence metrics, use over time, number of logins etc.), in addition to primary/secondary outcomes. (Note: Only report in the abstract what the main paper is reporting. If this information is missing from the main body of text, consider adding it)

subitem not at all important

1 ○

2 ○

3 ○

4 ◉

5 ○

essential

Limpar seleção

Does your paper address subitem 1b-iv?

Copy and paste relevant sections from the manuscript abstract (include quotes in quotation marks "like this" to indicate direct quotes from your manuscript), or elaborate on this item by providing additional information not in the ms, or briefly explain why the item is not applicable/relevant for your study

«"Be a Mom" was found to be effective in reducing depressive (...) and anxiety symptoms (...), in comparison to the control group where such changes were inexistent or much smaller. All the three psychological processes under study improved significantly in post-treatment assessments: emotion regulation ability (...) and psychological flexibility (...) improved only in the intervention group, and even if self-compassion increased in both groups (...), those improvements were considerably greater in the intervention group.»

Results section, "Participants": "Of the eligible women, 1058 (76.9%) completed baseline assessment and were randomized to the intervention (n = 545) or to the control (n =

1b-v) CONCLUSIONS/DISCUSSION in abstract for negative trials

Conclusions/Discussions in abstract for negative trials: Discuss the primary outcome - if the trial is negative (primary outcome not changed), and the intervention was not used, discuss whether negative results are attributable to lack of uptake and discuss reasons. (Note: Only report in the abstract what the main paper is reporting. If this information is missing from the main body of text, consider adding it)

subitem not at all important

1 ○

2 ○

3 ○

4 ●

5 ○

essential

Limpar seleção

Does your paper address subitem 1b-v?

Copy and paste relevant sections from the manuscript abstract (include quotes in quotation marks "like this" to indicate direct quotes from your manuscript), or elaborate on this item by providing additional information not in the ms, or briefly explain why the item is not applicable/relevant for your study

"Conclusions: These results suggest that “Be a Mom”, a low intensity cognitive behavior therapy program, is a promising first-line intervention for helping perinatal women, particularly those with early-onset PPD symptoms."

INTRODUCTION

2a) In INTRODUCTION: Scientific background and explanation of rationale

2a-i) Problem and the type of system/solution

Describe the problem and the type of system/solution that is object of the study: intended as stand-alone intervention vs. incorporated in broader health care program? Intended for a particular patient population? Goals of the intervention, e.g., being more cost-effective to other interventions, replace or complement other solutions? (Note: Details about the intervention are provided in “Methods” under 5)

subitem not at all important

1 ○

2 ○

3 ○

4 ○

5 ◉

essential

Limpar seleção

Does your paper address subitem 2a-i? *

Copy and paste relevant sections from the manuscript (include quotes in quotation marks "like this" to indicate direct quotes from your manuscript), or elaborate on this item by providing additional information not in the ms, or briefly explain why the item is not applicable/relevant for your study

"Even though PPD is treatable and amenable to preventive efforts, most women do not seek professional help for their perinatal depression symptoms [4], with recent research showing that attitudinal (e.g., thinking that no one will be able to help), knowledge (e.g., not knowing if one's problems are a reason to ask for help) and structural barriers (e.g., not having time or not being able to afford treatment) are the most common help-seeking barriers of perinatal women [5]. One increasingly popular approach of improving access to treatment is the utilization of web-based intervention programs, particularly low intensity interventions. These interventions offer several advantages over traditional formats of assistance, including greater temporal and local independency, anonymity, accessibility, and flexible delivery [6], making them particularly recommended for perinatal women.
Despite other web-based interventions have been developed for PPD [7], most of them were not designed or tested for preventive purposes (with the notable exceptions of "Mothers and Babies Course" [8] and "Sunnyside" [9], which nevertheless failed to provide compelling evidence on their efficacy), and they relied exclusively on classic cognitive-behavioral therapy [CBT] principles (e.g.,[10-11]), thus neglecting various concepts emphasized by "third wave" CBT (e.g., acceptance, cognitive defusion), which have been recently added to traditional CBT packages to foster intervention processes and outcomes [12]."

2a-ii) Scientific background, rationale: What is known about the (type of) system

Scientific background, rationale: What is known about the (type of) system that is the object of the study (be sure to discuss the use of similar systems for other conditions/diagnoses, if appropiate), motivation for the study, i.e. what are the reasons for and what is the context for this specific study, from which stakeholder viewpoint is the study performed, potential impact of findings [2]. Briefly justify the choice of the comparator.

subitem not at all important

1 ○

2 ○

3 ○

4 ○

5 ◉

essential

Limpar seleção

Does your paper address subitem 2a-ii? *

Copy and paste relevant sections from the manuscript (include quotes in quotation marks "like this" to indicate direct quotes from your manuscript), or elaborate on this item by providing additional information not in the ms, or briefly explain why the item is not applicable/relevant for your study

"Preliminary evidence from a pilot RCT attested the feasibility of the program and its effectiveness in reducing depression and anxiety symptoms of women at high-risk for PPD [15]. Furthermore, a greater decrease in depressive symptoms was found to be associated with a greater decrease in emotion regulation difficulties and a greater increase in self-compassion, while psychological flexibility was found to be unrelated to changes in depression levels [16].
(...)
Despite being the core the establishment of evidence-based interventions [12], the examination of mechanisms of change within the context of web-based interventions for PPD has not been performed until recently [16]."

2b) In INTRODUCTION: Specific objectives or hypotheses

Does your paper address CONSORT subitem 2b? *

Copy and paste relevant sections from the manuscript (include quotes in quotation marks "like this" to indicate direct quotes from your manuscript), or elaborate on this item by providing additional information not in the ms, or briefly explain why the item is not applicable/relevant for your study

"Given the fact that interventions to prevent PPD are most effective when they target at-risk women [17], this randomized controlled trial focused on a large sample of women at high-risk for PDD to ascertain the mechanisms of change associated with the efficacy of "Be a Mom" in reducing postpartum depression and anxiety symptoms, as they often occur in comorbidity. Specifically, bearing in mind that depression and anxiety are distinct but highly concurrent clinical phenomena [3], the study sought to examine whether changes in psychological processes (i.e., emotion regulation, self-compassion and psychological flexibility) were associated with changes in depressive and anxiety symptoms, among women who participated in the "Be a Mom" program (see Figure 1)."

METHODS

3a) Description of trial design (such as parallel, factorial) including allocation ratio

Does your paper address CONSORT subitem 3a? *

Copy and paste relevant sections from the manuscript (include quotes in quotation marks "like this" to indicate direct quotes from your manuscript), or elaborate on this item by providing additional information not in the ms, or briefly explain why the item is not applicable/relevant for your study

"This study was a two-arm, open-label randomized controlled trial to assess the efficacy of "Be a Mom" intervention, in comparison with a waiting-list control group receiving usual care, among women presenting high-risk for PPD. The trial is registered at ClinicalTrials.gov (NCT03024645). (...) Eligible women who consented to participate in the study were given baseline assessment (T0), using the online survey platform Limesurvey. After completing the baseline assessment, eligible participants were randomly assigned using a computerized random number generator (allocation rate: 1:1) to the intervention group ("Be a Mom" intervention) or to the control group (waiting-list group receiving usual care)."

3b) Important changes to methods after trial commencement (such as eligibility criteria), with reasons

Does your paper address CONSORT subitem 3b? *

Copy and paste relevant sections from the manuscript (include quotes in quotation marks "like this" to indicate direct quotes from your manuscript), or elaborate on this item by providing additional information not in the ms, or briefly explain why the item is not applicable/relevant for your study

No important changes to methods were made after trial commencement.

3b-i) Bug fixes, Downtimes, Content Changes

Bug fixes, Downtimes, Content Changes: ehealth systems are often dynamic systems. A description of changes to methods therefore also includes important changes made on the intervention or comparator during the trial (e.g., major bug fixes or changes in the functionality or content) (5-iii) and other “unexpected events” that may have influenced study design such as staff changes, system failures/downtimes, etc. [2].

subitem not at all important

1 ○

2 ○

3 ○

4 ◉

5 ○

essential

Limpar seleção

Does your paper address subitem 3b-i?

Copy and paste relevant sections from the manuscript (include quotes in quotation marks "like this" to indicate direct quotes from your manuscript), or elaborate on this item by providing additional information not in the ms, or briefly explain why the item is not applicable/relevant for your study

Not applicable; there were no bug fixes, downtimes or content changes within this RCT.

4a) Eligibility criteria for participants

Does your paper address CONSORT subitem 4a? *

Copy and paste relevant sections from the manuscript (include quotes in quotation marks "like this" to indicate direct quotes from your manuscript), or elaborate on this item by providing additional information not in the ms, or briefly explain why the item is not applicable/relevant for your study

Eligibility criteria to participate in the study were as follows: a) being an adult woman (≥18 years) in the early postpartum period (up to 3 months postpartum); b) presenting high-risk for PPD (a score of 5.5 or higher in the Postpartum Depression Predictors Inventory-Revised [PDPI-R; see "Measures"]; c) having a computer/tablet/smartphone and internet access at home; d) having the ability to read and speak Portuguese; and e) being a Portugal resident. Exclusion criteria were the presence of a severe medically diagnosed health condition in the infant or in the mother (e.g., cerebral palsy in the infant, or schizophrenia spectrum or other psychotic disorders in the mother), as self-reported by mothers. Those women who did not meet eligibility criteria were informed through email and were advised to seek professional help, if needed.

4a-i) Computer / Internet literacy

Computer / Internet literacy is often an implicit "de facto" eligibility criterion - this should be explicitly clarified.

subitem not at all important

1 ○

2 ○

3 ○

4 ○

5 ◉

essential

Limpar seleção

Does your paper address subitem 4a-i?

Copy and paste relevant sections from the manuscript (include quotes in quotation marks "like this" to indicate direct quotes from your manuscript), or elaborate on this item by providing additional information not in the ms, or briefly explain why the item is not applicable/relevant for your study

"Eligibility criteria to participate in the study were as follows: (...) c) having a computer/tablet/smartphone and internet access at home; d) having the ability to read and speak Portuguese; (...)"

4a-ii) Open vs. closed, web-based vs. face-to-face assessments:

Open vs. closed, web-based vs. face-to-face assessments: Mention how participants were recruited (online vs. offline), e.g., from an open access website or from a clinic, and clarify if this was a purely web-based trial, or there were face-to-face components (as part of the intervention or for assessment), i.e., to what degree got the study team to know the participant. In online-only trials, clarify if participants were quasi-anonymous and whether having multiple identities was possible or whether technical or logistical measures (e.g., cookies, email confirmation, phone calls) were used to detect/prevent these.

subitem not at all important

1 ○

2 ○

3 ○

4 ○

5 ◉

essential

Limpar seleção

Does your paper address subitem 4a-ii? *

Copy and paste relevant sections from the manuscript (include quotes in quotation marks "like this" to indicate direct quotes from your manuscript), or elaborate on this item by providing additional information not in the ms, or briefly explain why the item is not applicable/relevant for your study

"Recruitment occurred online between January 2019 and January 2021, both through unpaid cross-posting and paid advertisements on social media networks (Facebook and Instagram). Paid advertisements/campaigns targeted women aged 18-45 years old with interests in maternity topics, with the following tagline: "Did you have a baby in the last three months? We want to know if "Be a Mom" is effective in promoting postpartum women's mental health, and you can help us! To know if you are eligible to participate in the study fill out the following form and we will contact you." Before getting access to the eligibility form (including a set of questions to assess eligibility criteria and contact information), women were given information about the study goals and procedures (including voluntary participation and data protection issues), the participants' and researchers' roles were clarified, and women were asked their informed consent to participate in the study (by clicking the option "I understand and accept the conditions of the study")."

4a-iii) Information giving during recruitment

Information given during recruitment. Specify how participants were briefed for recruitment and in the informed consent procedures (e.g., publish the informed consent documentation as appendix, see also item X26), as this information may have an effect on user self-selection, user expectation and may also bias results.

subitem not at all important

1 ○

2 ○

3 ○

4 ○

5 ◉

essential

Limpar seleção

Does your paper address subitem 4a-iii?

Copy and paste relevant sections from the manuscript (include quotes in quotation marks "like this" to indicate direct quotes from your manuscript), or elaborate on this item by providing additional information not in the ms, or briefly explain why the item is not applicable/relevant for your study

"Before getting access to the eligibility form (including a set of questions to assess eligibility criteria and contact information), women were given information about the study goals and procedures (including voluntary participation and data protection issues), the participants' and researchers' roles were clarified, and women were asked their informed consent to participate in the study (by clicking the option "I understand and accept the conditions of the study")."

4b) Settings and locations where the data were collected

Does your paper address CONSORT subitem 4b? *

Copy and paste relevant sections from the manuscript (include quotes in quotation marks "like this" to indicate direct quotes from your manuscript), or elaborate on this item by providing additional information not in the ms, or briefly explain why the item is not applicable/relevant for your study

"Recruitment occurred online between January 2019 and January 2021, both through unpaid cross-posting and paid advertisements on social media networks (Facebook and Instagram).
(...)
Eligible women who consented to participate in the study were given baseline assessment (T0), using the online survey platform Limesurvey. (...) According to the guidelines established by the Pregnancy Surveillance Program in Portugal [20], postpartum usual care encompasses at least one medical appointment with a designated obstetrician or the woman's family doctor, and weekly visits to nursing services in primary care to monitor mothers' and their infants' general health status during the first two months postpartum."

4b-i) Report if outcomes were (self-)assessed through online questionnaires

Clearly report if outcomes were (self-)assessed through online questionnaires (as common in web-based trials) or otherwise.

subitem not at all important

1 ○

2 ○

3 ○

4 ○

5 ◉

essential

Limpar seleção

Does your paper address subitem 4b-i? *

Copy and paste relevant sections from the manuscript (include quotes in quotation marks "like this" to indicate direct quotes from your manuscript), or elaborate on this item by providing additional information not in the ms, or briefly explain why the item is not applicable/relevant for your study

"Eligible women who consented to participate in the study were given baseline assessment (T0), using the online survey platform Limesurvey. After completing the baseline assessment, eligible participants were randomly assigned using a computerized random number generator (allocation rate: 1:1) to the intervention group ("Be a Mom" intervention) or to the control group (waiting-list group receiving usual care).
(...) Post-intervention (T1) assessment was also performed online (through LimeSurvey platform) both in the intervention and control groups (after the intervention or eight weeks after randomization)."

4b-ii) Report how institutional affiliations are displayed

Report how institutional affiliations are displayed to potential participants [on ehealth media], as affiliations with prestigious hospitals or universities may affect volunteer rates, use, and reactions with regards to an intervention.(Not a required item – describe only if this may bias results)

subitem not at all important

1 ○

2 ○

3 ◉

4 ○

5 ○

essential

Limpar seleção

Does your paper address subitem 4b-ii?

Copy and paste relevant sections from the manuscript (include quotes in quotation marks "like this" to indicate direct quotes from your manuscript), or elaborate on this item by providing additional information not in the ms, or briefly explain why the item is not applicable/relevant for your study

The following information, quoted from the manuscript, was shared with the participants within their informed consent: "The study was approved by the Ethics Committee of the Faculty of Psychology and Educational Sciences, University of

5) The interventions for each group with sufficient details to allow replication, including how and when they were actually administered

5-i) Mention names, credential, affiliations of the developers, sponsors, and owners

Mention names, credential, affiliations of the developers, sponsors, and owners [6] (if authors/evaluators are owners or developer of the software, this needs to be declared in a “Conflict of interest” section or mentioned elsewhere in the manuscript).

subitem not at all important

1 ○

2 ○

3 ○

4 ○

5 ◉

essential

Limpar seleção

Does your paper address subitem 5-i?

Copy and paste relevant sections from the manuscript (include quotes in quotation marks "like this" to indicate direct quotes from your manuscript), or elaborate on this item by providing additional information not in the ms, or briefly explain why the item is not applicable/relevant for your study

«“Be a Mom” is a brief, self-guided, web-based selective/indicated preventive intervention that is primarily targeted at women who exhibit risk for PPD and/or present early-onset PPD symptoms. (...) The content of each module includes psychoeducational information combined with practical exercises and endorses the structured and goal-oriented nature of CBT sessions [14].»

5-ii) Describe the history/development process

Describe the history/development process of the application and previous formative evaluations (e.g., focus groups, usability testing), as these will have an impact on adoption/use rates and help with interpreting results.

subitem not at all important

1 ○

2 ○

3 ○

4 ○

5 ◉

essential

Limpar seleção

Does your paper address subitem 5-ii?

Copy and paste relevant sections from the manuscript (include quotes in quotation marks "like this" to indicate direct quotes from your manuscript), or elaborate on this item by providing additional information not in the ms, or briefly explain why the item is not applicable/relevant for your study

«“Be a Mom” is a brief, self-guided, web-based selective/indicated preventive intervention that is primarily targeted at women who exhibit risk for PPD and/or present early-onset PPD symptoms. (...) The content of each module includes psychoeducational information combined with practical exercises and endorses the structured and goal-oriented nature of CBT sessions [14].» (...) «Preliminary evidence from a pilot RCT attested the feasibility of the program and its effectiveness in reducing depression and anxiety symptoms of women at high-risk for PPD [15]. Furthermore, a greater decrease in depressive symptoms was found to be associated with a greater decrease in emotion regulation difficulties and a greater increase in self-compassion, while psychological flexibility was found to be unrelated to changes in depression levels

5-iii) Revisions and updating

Revisions and updating. Clearly mention the date and/or version number of the application/intervention (and comparator, if applicable) evaluated, or describe whether the intervention underwent major changes during the evaluation process, or whether the development and/or content was “frozen” during the trial. Describe dynamic components such as news feeds or changing content which may have an impact on the replicability of the intervention (for unexpected events see item 3b).

subitem not at all important

1 ○

2 ○

3 ○

4 ○

5 ◉

essential

Limpar seleção

Does your paper address subitem 5-iii?

Copy and paste relevant sections from the manuscript (include quotes in quotation marks "like this" to indicate direct quotes from your manuscript), or elaborate on this item by providing additional information not in the ms, or briefly explain why the item is not applicable/relevant for your study

Not applicable; no revisions or updating were performed during the RCT.

5-iv) Quality assurance methods

Provide information on quality assurance methods to ensure accuracy and quality of information provided [1], if applicable.

subitem not at all important

1 ○

2 ○

3 ○

4 ○

5 ◉

essential

Limpar seleção

Does your paper address subitem 5-iv?

Copy and paste relevant sections from the manuscript (include quotes in quotation marks "like this" to indicate direct quotes from your manuscript), or elaborate on this item by providing additional information not in the ms, or briefly explain why the item is not applicable/relevant for your study

"Email reminders were sent automatically to the participants if they went 3, 7 and 13 days without accessing it. The periodicity of the reminders was defined according to the guidelines provided by the software company that developed the web-based program and based on previous feedback obtained from postpartum women during the pilot RCT [15]. Noteworthy, the sequence of periodical reminders restarted every time a woman returned to proceed with the program, i.e., they were unrelated to the amount of content that had been already completed. Asynchronous communication channels were provided for program-related support only. Access to the program was free of cost, and no compensation was given to participants. Participants assigned to the control group were offered no intervention but were free to access usual care (as were all participants). At the end of the RCT (including 3-months and 12-months follow-up assessments), they were offered access to “Be a Mom” intervention. Given that the first three to four months postpartum seem to be a high-risk period for depression [21], women in the wait-list control group were initially informed about their risk for PPD and instructed to interrupt their participation in the RCT and to seek professional help within the usual care if they felt their health condition was worsening."

5-v) Ensure replicability by publishing the source code, and/or providing screenshots/screen-capture video, and/or providing flowcharts of the algorithms used

Ensure replicability by publishing the source code, and/or providing screenshots/screen-capture video, and/or providing flowcharts of the algorithms used. Replicability (i.e., other researchers should in principle be able to replicate the study) is a hallmark of scientific reporting.

subitem not at all important

1 ○

2 ○

3 ○

4 ○

5 ◉

essential

Limpar seleção

Does your paper address subitem 5-v?

Copy and paste relevant sections from the manuscript (include quotes in quotation marks "like this" to indicate direct quotes from your manuscript), or elaborate on this item by providing additional information not in the ms, or briefly explain why the item is not applicable/relevant for your study

Figure 1. Conceptual diagram of the study.
Figure 2. Univariate Latent Change Score model representation.
Figure 3. Participants’ Flow Diagram.

5-vi) Digital preservation

Digital preservation: Provide the URL of the application, but as the intervention is likely to change or disappear over the course of the years; also make sure the intervention is archived (Internet Archive, webcitation.org, and/or publishing the source code or screenshots/videos alongside the article). As pages behind login screens cannot be archived, consider creating demo pages which are accessible without login.

subitem not at all important

1 ○

2 ○

3 ○

4 ○

5 ◉

essential

Limpar seleção

Does your paper address subitem 5-vi?

Copy and paste relevant sections from the manuscript (include quotes in quotation marks "like this" to indicate direct quotes from your manuscript), or elaborate on this item by providing additional information not in the ms, or briefly explain why the item is not applicable/relevant for your study

This is the original, permanente URL for the app: https://beamom.pt/; the English version is under progress.

5-vii) Access

Access: Describe how participants accessed the application, in what setting/context, if they had to pay (or were paid) or not, whether they had to be a member of specific group. If known, describe how participants obtained "access to the platform and Internet" [1]. To ensure access for editors/reviewers/readers, consider to provide a "backdoor" login account or demo mode for reviewers/readers to explore the application (also important for archiving purposes, see vi).

subitem not at all important

1 ○

2 ○

3 ○

4 ○

5 ◉

essential

Limpar seleção

Does your paper address subitem 5-vii? *

Copy and paste relevant sections from the manuscript (include quotes in quotation marks "like this" to indicate direct quotes from your manuscript), or elaborate on this item by providing additional information not in the ms, or briefly explain why the item is not applicable/relevant for your study

"Women assigned to the intervention arm were invited by email to register in a password-protected website that contained the “Be a Mom” intervention (beamom.pt; access to the program was restricted to invitation). Approximately two weeks after registration, participants were contacted via telephone by the research team to assess if there were any difficulties in accessing the website and to clarify any questions concerning the program’s flow and functioning. After registration, women have access to the 5 modules (the “Couple’s Relationship” module is only presented to women in a relationship) and were instructed that they should complete one module per week following the module’s order, although a slower pace is allowed. Each module has an approximate length of 45 minutes. Women were given the option of pausing the module and resuming the last page visited during subsequent access. Although it was generally recommended to complete one module per week, given the usual competing demands of the postpartum period, women were allowed to complete the program within a maximum period of 8 weeks. Email reminders were sent automatically to the participants if they went 3, 7 and 13 days without accessing it. The periodicity of the reminders was defined according to the guidelines provided by the software company that developed the web-based program and based on previous feedback obtained from postpartum women during the pilot RCT [15]. Noteworthy, the sequence of periodical reminders restarted every time a woman returned to proceed with the program, i.e., they were unrelated to the amount of content that had been already completed. Asynchronous communication channels were provided for program-related support only. Access to the program was free of cost, and no compensation was given to participants. Participants assigned to the control group were offered no intervention but were free to access usual care (as were all participants). At the end of the RCT (including 3-months and 12-months follow-up assessments), they were offered access

5-viii) Mode of delivery, features/functionalities/components of the intervention and comparator, and the theoretical framework

Describe mode of delivery, features/functionalities/components of the intervention and comparator, and the theoretical framework [6] used to design them (instructional strategy [1], behaviour change techniques, persuasive features, etc., see e.g., [7, 8] for terminology). This includes an in-depth description of the content (including where it is coming from and who developed it) [1],” whether [and how] it is tailored to individual circumstances and allows users to track their progress and receive feedback” [6]. This also includes a description of communication delivery channels and – if computer-mediated communication is a component – whether communication was synchronous or asynchronous [6]. It also includes information on presentation strategies [1], including page design principles, average amount of text on pages, presence of hyperlinks to other resources, etc. [1].

subitem not at all important

- 1 ○
- 2 ○
- 3 ○
- 4 ○
- 5 ◉

essential

Limpar seleção

Does your paper address subitem 5-viii? *

Copy and paste relevant sections from the manuscript (include quotes in quotation marks "like this" to indicate direct quotes from your manuscript), or elaborate on this item by providing additional information not in the ms, or briefly explain why the item is not applicable/relevant for your study

«“Be a Mom” is a brief, self-guided, web-based selective/indicated preventive intervention that is primarily targeted at women who exhibit risk for PPD and/or present early-onset PPD symptoms. Grounded in CBT principles, it combines the classic CBT approach (e.g., cognitive restructuring, activity scheduling) with more recent “third wave” CBT features (e.g., values clarification, self-compassion). “Be a Mom” is
a structured program with a modular approach that encompasses five sequential modules on the following topics: (1) “Changes and Emotional Reactions” (...); (2) “Cognitions” (...); (3) “Values and Social Support” (...); (4) “Couple Relationship” (...); and (5) “PPD Alert Signs and Professional Help-seeking” (...). The content of each module includes psychoeducational information combined with practical exercises and endorses the structured and goal-oriented nature of CBT sessions [14].»

5-ix) Describe use parameters

Describe use parameters (e.g., intended “doses” and optimal timing for use). Clarify what instructions or recommendations were given to the user, e.g., regarding timing, frequency, heaviness of use, if any, or was the intervention used ad libitum.

subitem not at all important

1 ○

2 ○

3 ○

4 ○

5 ◉

essential

Limpar seleção

Does your paper address subitem 5-ix?

Copy and paste relevant sections from the manuscript (include quotes in quotation marks "like this" to indicate direct quotes from your manuscript), or elaborate on this item by providing additional information not in the ms, or briefly explain why the item is not applicable/relevant for your study

"Each module has an approximate length of 45 minutes. Women were given the option of pausing the module and resuming the last page visited during subsequent access. Although it was generally recommended to complete one module per week, given the usual competing demands of the postpartum period, women were allowed to complete the program within a maximum period of 8 weeks. Email reminders were sent automatically to the participants if they went 3, 7 and 13 days without accessing it. The periodicity of the reminders was defined according to the guidelines provided by the software company that developed the web-based program and based on previous feedback obtained from postpartum women during the pilot RCT [15]. Noteworthy, the sequence of periodical reminders restarted every time a woman returned to proceed with the program, i.e., they were unrelated to the amount of content that had been already completed. Asynchronous communication channels were provided for program-related support only. Access to the program was free of cost, and no compensation was given to participants. Participants assigned to the control group were offered no intervention but were free to access usual care (as were all participants). At the end of the RCT (including 3-months and 12-months follow-up assessments), they were offered access to "Be a Mom" intervention."

5-x) Clarify the level of human involvement

Clarify the level of human involvement (care providers or health professionals, also technical assistance) in the e-intervention or as co-intervention (detail number and expertise of professionals involved, if any, as well as “type of assistance offered, the timing and frequency of the support, how it is initiated, and the medium by which the assistance is delivered”. It may be necessary to distinguish between the level of human involvement required for the trial, and the level of human involvement required for a routine application outside of a RCT setting (discuss under item 21 – generalizability).

subitem not at all important

- 1 ○
- 2 ○
- 3 ○
- 4 ◉
- 5 ○

essential

Limpar seleção

Does your paper address subitem 5-x?

Copy and paste relevant sections from the manuscript (include quotes in quotation marks "like this" to indicate direct quotes from your manuscript), or elaborate on this item by providing additional information not in the ms, or briefly explain why the item is not applicable/relevant for your study

"One of the researchers was responsible for randomization, while other two researchers were responsible for the participants’ enrollment and groups assignment."

5-xi) Report any prompts/reminders used

Report any prompts/reminders used: Clarify if there were prompts (letters, emails, phone calls, SMS) to use the application, what triggered them, frequency etc. It may be necessary to distinguish between the level of prompts/reminders required for the trial, and the level of prompts/reminders for a routine application outside of a RCT setting (discuss under item 21 – generalizability).

subitem not at all important

1 ○

2 ○

3 ○

4 ○

5 ●

essential

Limpar seleção

Does your paper address subitem 5-xi? *

Copy and paste relevant sections from the manuscript (include quotes in quotation marks "like this" to indicate direct quotes from your manuscript), or elaborate on this item by providing additional information not in the ms, or briefly explain why the item is not applicable/relevant for your study

"After registration, women have access to the 5 modules (the "Couple's Relationship" module is only presented to women in a relationship) and were instructed that they should complete one module per week following the module's order, although a slower pace is allowed. Each module has an approximate length of 45 minutes. Women were given the option of pausing the module and resuming the last page visited during subsequent access. Although it was generally recommended to complete one module per week, given the usual competing demands of the postpartum period, women were allowed to complete the program within a maximum period of 8 weeks. Email reminders were sent automatically to the participants if they went 3, 7 and 13 days without accessing it. The periodicity of the reminders was defined according to the guidelines provided by the software company that developed the web-based program and based on previous feedback obtained from postpartum women during the pilot RCT [15]. Noteworthy, the sequence of periodical reminders restarted every time a woman returned to proceed with the program, i.e., they were unrelated to the amount of content that had been already completed."

5-xii) Describe any co-interventions (incl. training/support)

Describe any co-interventions (incl. training/support): Clearly state any interventions that are provided in addition to the targeted eHealth intervention, as ehealth intervention may not be designed as stand-alone intervention. This includes training sessions and support [1]. It may be necessary to distinguish between the level of training required for the trial, and the level of training for a routine application outside of a RCT setting (discuss under item 21 – generalizability.

subitem not at all important

1 ○

2 ○

3 ○

4 ○

5 ◉

essential

Limpar seleção

Does your paper address subitem 5-xii? *

Copy and paste relevant sections from the manuscript (include quotes in quotation marks "like this" to indicate direct quotes from your manuscript), or elaborate on this item by providing additional information not in the ms, or briefly explain why the item is not applicable/relevant for your study

"Eligible women who consented to participate in the study were given baseline assessment (T0), using the online survey platform Limesurvey. After completing the baseline assessment, eligible participants were randomly assigned using a computerized random number generator (allocation rate: 1:1) to the intervention group ("Be a Mom" intervention) or to the control group (waiting-list group receiving usual care). According to the guidelines established by the Pregnancy Surveillance Program in Portugal [20], postpartum usual care encompasses at least one medical appointment with a designated obstetrician or the woman's family doctor, and weekly visits to nursing services in primary care to monitor mothers' and their infants' general health status during the first two months postpartum.
(...)
Participants were informed about their assigned group through email (no blinding to the assigned group) and instructed to seek no other intervention besides "Be a Mom" and/or the usual care while participating in the RCT. "

6a) Completely defined pre-specified primary and secondary outcome measures, including how and when they were assessed

Does your paper address CONSORT subitem 6a? *

Copy and paste relevant sections from the manuscript (include quotes in quotation marks "like this" to indicate direct quotes from your manuscript), or elaborate on this item by providing additional information not in the ms, or briefly explain why the item is not applicable/relevant for your study

"Eligible women who consented to participate in the study were given baseline assessment (T0), using the online survey platform Limesurvey.
8...) Post-intervention (T1) assessment was also performed online (through LimeSurvey platform) both in the intervention and control groups (after the intervention or eight weeks after randomization). (...) To examine changes over time both in the adjustment outcomes (depressive and anxiety symptoms) and in the psychological processes (emotion regulation, self-compassion and psychological flexibility), univariate LCS models were computed [29]."

6a-i) Online questionnaires: describe if they were validated for online use and apply CHERRIES items to describe how the questionnaires were designed/deployed

If outcomes were obtained through online questionnaires, describe if they were validated for online use and apply CHERRIES items to describe how the questionnaires were designed/deployed [9].

subitem not at all important

1 ○

2 ○

3 ○

4 ◉

5 ○

essential

Limpar seleção

Does your paper address subitem 6a-i?

Copy and paste relevant sections from manuscript text

Although the questionnaires were not specifically validated for online use, it is implicit that they had been successfully used in previous studies with this app:
" Preliminary evidence from a pilot RCT attested the feasibility of the program and its effectiveness in reducing depression and anxiety symptoms of women at high-risk for PPD [15]. Furthermore, a greater decrease in depressive symptoms was found to be associated with a greater decrease in emotion regulation difficulties and a greater increase in self-compassion, while psychological flexibility was found to be unrelated to changes in depression levels [16]."

6a-ii) Describe whether and how “use” (including intensity of use/dosage) was defined/measured/monitored

Describe whether and how “use” (including intensity of use/dosage) was defined/measured/monitored (logins, logfile analysis, etc.). Use/adoption metrics are important process outcomes that should be reported in any ehealth trial.

subitem not at all important

1 ○

2 ○

3 ○

4 ○

5 ◉

essential

Limpar seleção

Does your paper address subitem 6a-ii?

Copy and paste relevant sections from manuscript text

"After registration, women have access to the 5 modules (the “Couple’s Relationship” module is only presented to women in a relationship) and were instructed that they should complete one module per week following the module’s order, although a slower pace is allowed. Each module has an approximate length of 45 minutes. Women were given the option of pausing the module and resuming the last page visited during subsequent access. Although it was generally recommended to complete one module per week, given the usual competing demands of the postpartum period, women were allowed to complete the program within a maximum period of 8 weeks. Email reminders were sent automatically to the participants if they went 3, 7 and 13 days without accessing it. The periodicity of the reminders was defined according to the guidelines provided by the software company that developed the web-based program and based on previous feedback obtained from postpartum women during the pilot RCT [15]. Noteworthy, the sequence of periodical reminders restarted every time a woman returned to proceed with the program, i.e., they were unrelated to the amount of content that had been already completed."

6a-iii) Describe whether, how, and when qualitative feedback from participants was obtained

Describe whether, how, and when qualitative feedback from participants was obtained (e.g., through emails, feedback forms, interviews, focus groups).

subitem not at all important

1 ○

2 ○

3 ○

4 ○

5 ◉

essential

Limpar seleção

Does your paper address subitem 6a-iii?

Copy and paste relevant sections from manuscript text

"The periodicity of the reminders was defined according to the guidelines provided by the software company that developed the web-based program and based on previous feedback obtained from postpartum women during the pilot RCT [15]."

14. Fonseca, A, Pereira, M, Araújo-Pedrosa, A, Gorayeb, R, Ramos, MM, & Canavarro, MC. Be a Mom: Formative evaluation of a web-based psychological intervention to prevent postpartum depression. Cognitive and Behavioral Practice; 2018,25(4):473-495. doi:10.1016/j.cbpra.2018.02.002
15. Fonseca, A, Alves, S, Monteiro, F, Gorayeb, R, Canavarro, MC. “Be a Mom”, a web-based intervention to prevent postpartum depression: Results from a pilot randomized controlled trial. Behavior Therapy; 2020,51(4):616-633. doi:10.1016/j.beth.2019.09.007

6b) Any changes to trial outcomes after the trial commenced, with reasons

Does your paper address CONSORT subitem 6b? *

Copy and paste relevant sections from the manuscript (include quotes in quotation marks "like this" to indicate direct quotes from your manuscript), or elaborate on this item by providing additional information not in the ms, or briefly explain why the item is not applicable/relevant for your study

Not applicable; there were no changes to trial outcomes after the trial had commenced.

7a) How sample size was determined

NPT: When applicable, details of whether and how the clustering by care provides or centers was addressed

7a-i) Describe whether and how expected attrition was taken into account when calculating the sample size

Describe whether and how expected attrition was taken into account when calculating the sample size.

subitem not at all important

1 ○

2 ○

3 ○

4 ◉

5 ○

essential

Limpar seleção

Does your paper address subitem 7a-i?

Copy and paste relevant sections from manuscript title (include quotes in quotation marks "like this" to indicate direct quotes from your manuscript), or elaborate on this item by providing additional information not in the ms, or briefly explain why the item is not applicable/relevant for your study

"To reduce attrition, weekly reminders (email and text messages on an alternate basis) were sent during one month to women who failed to complete baseline and post-intervention assessments. (...) Of the 2367 women enrolled in the study between January 2019 and January 2021, 1980 were assessed concerning risk for PPD, and 1376 women who presented high-risk for PPD (69.5% of women) were given baseline assessment. Of the eligible women, 1058 (76.9%) completed baseline assessment and were randomized to the intervention (n = 545) or to the control (n = 513) conditions."

7b) When applicable, explanation of any interim analyses and stopping guidelines

Does your paper address CONSORT subitem 7b? *

Copy and paste relevant sections from the manuscript (include quotes in quotation marks "like this" to indicate direct quotes from your manuscript), or elaborate on this item by providing additional information not in the ms, or briefly explain why the item is not applicable/relevant for your study

Not applicable; this RCT did not use stopping guidelines, nor encompassed any interim analyses of the accumulating data.

8a) Method used to generate the random allocation sequence

NPT: When applicable, how care providers were allocated to each trial group

Does your paper address CONSORT subitem 8a? *

Copy and paste relevant sections from the manuscript (include quotes in quotation marks "like this" to indicate direct quotes from your manuscript), or elaborate on this item by providing additional information not in the ms, or briefly explain why the item is not applicable/relevant for your study

"After completing the baseline assessment, eligible participants were randomly assigned using a computerized random number generator (allocation rate: 1:1) to the intervention group ("Be a Mom" intervention) or to the control group (waiting-list group receiving usual care)."

8b) Type of randomisation; details of any restriction (such as blocking and block size)

Does your paper address CONSORT subitem 8b? *

Copy and paste relevant sections from the manuscript (include quotes in quotation marks "like this" to indicate direct quotes from your manuscript), or elaborate on this item by providing additional information not in the ms, or briefly explain why the item is not applicable/relevant for your study

"This study was a two-arm, open-label randomized controlled trial to assess the efficacy of “Be a Mom” intervention, in comparison with a waiting-list control group receiving usual care, among women presenting high-risk for PPD."
"After completing the baseline assessment, eligible participants were randomly assigned using a computerized random number generator (allocation rate: 1:1) to the intervention group (“Be a Mom” intervention) or to the control group (waiting-list group receiving usual care)."

9) Mechanism used to implement the random allocation sequence (such as sequentially numbered containers), describing any steps taken to conceal the sequence until interventions were assigned

Does your paper address CONSORT subitem 9? *

Copy and paste relevant sections from the manuscript (include quotes in quotation marks "like this" to indicate direct quotes from your manuscript), or elaborate on this item by providing additional information not in the ms, or briefly explain why the item is not applicable/relevant for your study

"After completing the baseline assessment, eligible participants were randomly assigned using a computerized random number generator (allocation rate: 1:1) to the intervention group (“Be a Mom” intervention) or to the control group (waiting-list group receiving usual care)."

10) Who generated the random allocation sequence, who enrolled participants, and who assigned participants to interventions

Does your paper address CONSORT subitem 10? *

Copy and paste relevant sections from the manuscript (include quotes in quotation marks "like this" to indicate direct quotes from your manuscript), or elaborate on this item by providing additional information not in the ms, or briefly explain why the item is not applicable/relevant for your study

"One of the researchers was responsible for randomization, while other two researchers were responsible for the participants' enrollment and groups assignment."

11a) If done, who was blinded after assignment to interventions (for example, participants, care providers, those assessing outcomes) and how

NPT: Whether or not administering co-interventions were blinded to group assignment

11a-i) Specify who was blinded, and who wasn't

Specify who was blinded, and who wasn't. Usually, in web-based trials it is not possible to blind the participants [1, 3] (this should be clearly acknowledged), but it may be possible to blind outcome assessors, those doing data analysis or those administering co-interventions (if any).

subitem not at all important

- 1 ○
- 2 ○
- 3 ○
- 4 ○
- 5 ◉

essential

Limpar seleção

Does your paper address subitem 11a-i? *

Copy and paste relevant sections from the manuscript (include quotes in quotation marks "like this" to indicate direct quotes from your manuscript), or elaborate on this item by providing additional information not in the ms, or briefly explain why the item is not applicable/relevant for your study

"Participants were informed about their assigned group through email (no blinding to the assigned group) and instructed to seek no other intervention besides “Be a Mom” and/or the usual care while participating in the RCT."

11a-ii) Discuss e.g., whether participants knew which intervention was the “intervention of interest” and which one was the “comparator”

Informed consent procedures (4a-ii) can create biases and certain expectations - discuss e.g., whether participants knew which intervention was the “intervention of interest” and which one was the “comparator”.

subitem not at all important

- 1 ○
- 2 ○
- 3 ○
- 4 ○
- 5 ◉

essential

Limpar seleção

Does your paper address subitem 11a-ii?

Copy and paste relevant sections from the manuscript (include quotes in quotation marks "like this" to indicate direct quotes from your manuscript), or elaborate on this item by providing additional information not in the ms, or briefly explain why the item is not applicable/relevant for your study

"Participants were informed about their assigned group through email (no blinding to the assigned group) and instructed to seek no other intervention besides “Be a Mom” and/or the usual care while participating in the RCT. (...) Participants assigned to the control group were offered no intervention but were free to access usual care (as were all participants). At the end of the RCT (including 3-months and 12-months follow-up assessments), they were offered access to “Be a Mom” intervention."

11b) If relevant, description of the similarity of interventions

(this item is usually not relevant for ehealth trials as it refers to similarity of a placebo or sham intervention to a active medication/intervention)

Does your paper address CONSORT subitem 11b? *

Copy and paste relevant sections from the manuscript (include quotes in quotation marks "like this" to indicate direct quotes from your manuscript), or elaborate on this item by providing additional information not in the ms, or briefly explain why the item is not applicable/relevant for your study

The issue of similarity of interventions is not applicable to this RCT.

12a) Statistical methods used to compare groups for primary and secondary outcomes

NPT: When applicable, details of whether and how the clustering by care providers or centers was addressed

Does your paper address CONSORT subitem 12a? *

Copy and paste relevant sections from the manuscript (include quotes in quotation marks "like this" to indicate direct quotes from your manuscript), or elaborate on this item by providing additional information not in the ms, or briefly explain why the item is not applicable/relevant for your study

"To examine changes over time both in the adjustment outcomes (depressive and anxiety symptoms) and in the psychological processes (emotion regulation, self-compassion and psychological flexibility), univariate LCS models were computed [29].
(...)
Finally, to examine whether changes in psychological processes (emotion regulation, self-compassion and psychological flexibility) were associated with changes in women's adjustment outcomes (depressive and anxiety symptoms), a two-wave latent change scores model (2W-LCS) was conducted ([30, 33-34]."

12a-i) Imputation techniques to deal with attrition / missing values

Imputation techniques to deal with attrition / missing values: Not all participants will use the intervention/comparator as intended and attrition is typically high in ehealth trials. Specify how participants who did not use the application or dropped out from the trial were treated in the statistical analysis (a complete case analysis is strongly discouraged, and simple imputation techniques such as LOCF may also be problematic [4]).

subitem not at all important

1 ○

2 ○

3 ○

4 ○

5 ◉

essential

Limpar seleção

Does your paper address subitem 12a-i? *

Copy and paste relevant sections from the manuscript (include quotes in quotation marks "like this" to indicate direct quotes from your manuscript), or elaborate on this item by providing additional information not in the ms, or briefly explain why the item is not applicable/relevant for your study

"Missing data were handled using the Full Information Maximum Likelihood estimation [illegible]

12b) Methods for additional analyses, such as subgroup analyses and adjusted analyses

Does your paper address CONSORT subitem 12b? *

Copy and paste relevant sections from the manuscript (include quotes in quotation marks "like this" to indicate direct quotes from your manuscript), or elaborate on this item by providing additional information not in the ms, or briefly explain why the item is not applicable/relevant for your study

"A multigroup model approach was used to check for differences between the intervention and the control groups in LCS models (...)"

X26) REB/IRB Approval and Ethical Considerations [recommended as subheading under "Methods"] (not a CONSORT item)

X26-i) Comment on ethics committee approval

subitem not at all important

1 ○

2 ○

3 ○

4 ○

5 ◉

essential

Limpar seleção

Does your paper address subitem X26-i?

Copy and paste relevant sections from the manuscript (include quotes in quotation marks "like this" to indicate direct quotes from your manuscript), or elaborate on this item by providing additional information not in the ms, or briefly explain why the item is not applicable/relevant for your study

"The study was approved by the Ethics Committee of Faculty of Psychology and Educational Sciences, University of Coimbra."

x26-ii) Outline informed consent procedures

Outline informed consent procedures e.g., if consent was obtained offline or online (how? Checkbox, etc.?), and what information was provided (see 4a-ii). See [6] for some items to be included in informed consent documents.

subitem not at all important

1 ○

2 ○

3 ○

4 ○

5 ◉

essential

Limpar seleção

Does your paper address subitem X26-ii?

Copy and paste relevant sections from the manuscript (include quotes in quotation marks "like this" to indicate direct quotes from your manuscript), or elaborate on this item by providing additional information not in the ms, or briefly explain why the item is not applicable/relevant for your study

"Before getting access to the eligibility form (including a set of questions to assess eligibility criteria and contact information), women were given information about the study goals and procedures (including voluntary participation and data protection issues), the participants' and researchers' roles were clarified, and women were asked their informed consent to participate in the study (by clicking the option "I understand and accept the conditions of the study")."

X26-iii) Safety and security procedures

Safety and security procedures, incl. privacy considerations, and any steps taken to reduce the likelihood or detection of harm (e.g., education and training, availability of a hotline)

subitem not at all important

1 ○

2 ○

3 ○

4 ○

5 ●

essential

Limpar seleção

Does your paper address subitem X26-iii?

Copy and paste relevant sections from the manuscript (include quotes in quotation marks "like this" to indicate direct quotes from your manuscript), or elaborate on this item by providing additional information not in the ms, or briefly explain why the item is not applicable/relevant for your study

"Asynchronous communication channels were provided for program-related support only.
Given that the first three to four months postpartum seem to be a high-risk period for depression [21], women in the wait-list control group were initially informed about their risk for PPD and instructed to interrupt their participation in the RCT and to seek

RESULTS

13a) For each group, the numbers of participants who were randomly assigned, received intended treatment, and were analysed for the primary outcome

NPT: The number of care providers or centers performing the intervention in each group and the number of patients treated by each care provider in each center

Does your paper address CONSORT subitem 13a? *

Copy and paste relevant sections from the manuscript (include quotes in quotation marks "like this" to indicate direct quotes from your manuscript), or elaborate on this item by providing additional information not in the ms, or briefly explain why the item is not applicable/relevant for your study

Table 2. Changes in adjustment outcomes and psychological processes over time in the intervention and control groups.

13b) For each group, losses and exclusions after randomisation, together with reasons

Does your paper address CONSORT subitem 13b? (NOTE: Preferably, this is shown in a CONSORT flow diagram) *

Copy and paste relevant sections from the manuscript (include quotes in quotation marks "like this" to indicate direct quotes from your manuscript), or elaborate on this item by providing additional information not in the ms, or briefly explain why the item is not applicable/relevant for your study

Figure 3. Participants' Flow Diagram.

13b-i) Attrition diagram

Strongly recommended: An attrition diagram (e.g., proportion of participants still logging in or using the intervention/comparator in each group plotted over time, similar to a survival curve) or other figures or tables demonstrating usage/dose/engagement.

subitem not at all important

1 ○

2 ○

3 ○

4 ◉

5 ○

essential

Limpar seleção

Does your paper address subitem 13b-i?

Copy and paste relevant sections from the manuscript or cite the figure number if applicable (include quotes in quotation marks "like this" to indicate direct quotes from your manuscript), or elaborate on this item by providing additional information not in the ms, or briefly explain why the item is not applicable/relevant for your study

Please see: Figure 3 - Participants' Flow Diagram.

14a) Dates defining the periods of recruitment and follow-up

Does your paper address CONSORT subitem 14a? *

Copy and paste relevant sections from the manuscript (include quotes in quotation marks "like this" to indicate direct quotes from your manuscript), or elaborate on this item by providing additional information not in the ms, or briefly explain why the item is not applicable/relevant for your study

"Recruitment occurred online between January 2019 and January 2021 (...) At the end of the RCT (including 3-months and 12-months follow-up assessments), they were offered access to “Be a Mom” intervention."

14a-i) Indicate if critical “secular events” fell into the study period

Indicate if critical “secular events” fell into the study period, e.g., significant changes in Internet resources available or “changes in computer hardware or Internet delivery resources”

subitem not at all important

1 ○

2 ○

3 ○

4 ○

5 ◉

essential

Limpar seleção

Does your paper address subitem 14a-i?

Copy and paste relevant sections from the manuscript (include quotes in quotation marks "like this" to indicate direct quotes from your manuscript), or elaborate on this item by providing additional information not in the ms, or briefly explain why the item is not applicable/relevant for your study

There were no critical “secular events” fell into the study period.

14b) Why the trial ended or was stopped (early)

Does your paper address CONSORT subitem 14b? *

Copy and paste relevant sections from the manuscript (include quotes in quotation marks "like this" to indicate direct quotes from your manuscript), or elaborate on this item by providing additional information not in the ms, or briefly explain why the item is not applicable/relevant for your study

The trial did not end or stopped earlier than planned.

15) A table showing baseline demographic and clinical characteristics for each group

NPT: When applicable, a description of care providers (case volume, qualification, expertise, etc.) and centers (volume) in each group

Does your paper address CONSORT subitem 15? *

Copy and paste relevant sections from the manuscript (include quotes in quotation marks "like this" to indicate direct quotes from your manuscript), or elaborate on this item by providing additional information not in the ms, or briefly explain why the item is not applicable/relevant for your study

Table 1. Baseline sociodemographic and clinical characteristics: Comparison between the intervention and the control groups.

15-i) Report demographics associated with digital divide issues

In ehealth trials it is particularly important to report demographics associated with digital divide issues, such as age, education, gender, social-economic status, computer/Internet/ehealth literacy of the participants, if known.

subitem not at all important

1 ○

2 ○

3 ○

4 ○

5 ◉

essential

Limpar seleção

Does your paper address subitem 15-i? *

Copy and paste relevant sections from the manuscript (include quotes in quotation marks "like this" to indicate direct quotes from your manuscript), or elaborate on this item by providing additional information not in the ms, or briefly explain why the item is not applicable/relevant for your study

Table 1. Baseline sociodemographic and clinical characteristics: Comparison between the intervention and the control groups.

16) For each group, number of participants (denominator) included in each analysis and whether the analysis was by original assigned groups

16-i) Report multiple “denominators” and provide definitions

Report multiple “denominators” and provide definitions: Report N’s (and effect sizes) “across a range of study participation [and use] thresholds” [1], e.g., N exposed, N consented, N used more than x times, N used more than y weeks, N participants “used” the intervention/comparator at specific pre-defined time points of interest (in absolute and relative numbers per group). Always clearly define “use” of the intervention.

subitem not at all important

1 ○

2 ○

3 ○

4 ○

5 ◉

essential

Limpar seleção

Does your paper address subitem 16-i? *

Copy and paste relevant sections from the manuscript (include quotes in quotation marks "like this" to indicate direct quotes from your manuscript), or elaborate on this item by providing additional information not in the ms, or briefly explain why the item is not applicable/relevant for your study

"Of the eligible women, 1058 (76.9%) completed baseline assessment and were randomized to the intervention (n = 545) or to the control (n = 513) conditions." (...) "The 2W-LCS allows the estimation of the univariate LCS for each variable (...) and of the change-to-change effects (i.e., the estimate of the effect of the change in one variable on the change of the other variable) of psychological processes (...) on adjustment outcomes."

16-ii) Primary analysis should be intent-to-treat

Primary analysis should be intent-to-treat, secondary analyses could include comparing only “users”, with the appropriate caveats that this is no longer a randomized sample (see 18-i).

subitem not at all important

1 ○

2 ○

3 ○

4 ○

5 ◉

essential

Limpar seleção

Does your paper address subitem 16-ii?

Copy and paste relevant sections from the manuscript (include quotes in quotation marks "like this" to indicate direct quotes from your manuscript), or elaborate on this item by providing additional information not in the ms, or briefly explain why the item is not applicable/relevant for your study

"In addition to the main analyses, the same analyses were repeated in two subgroups of women: evaluative respondents (i.e., intention-to-treat analysis, by including women who completed both baseline and post-intervention assessments in the statistical analyses) and women presenting clinically relevant depressive symptoms, i.e., EPDS scores at baseline >9."

17a) For each primary and secondary outcome, results for each group, and the estimated effect size and its precision (such as 95% confidence interval)

Does your paper address CONSORT subitem 17a? *

Copy and paste relevant sections from the manuscript (include quotes in quotation marks "like this" to indicate direct quotes from your manuscript), or elaborate on this item by providing additional information not in the ms, or briefly explain why the item is not applicable/relevant for your study

Table 3. Two-wave latent change score models estimates: correlations at baseline, cross-lagged effects, and change-to-change effects in the intervention group and in the control group.

17a-i) Presentation of process outcomes such as metrics of use and intensity of use

In addition to primary/secondary (clinical) outcomes, the presentation of process outcomes such as metrics of use and intensity of use (dose, exposure) and their operational definitions is critical. This does not only refer to metrics of attrition (13-b) (often a binary variable), but also to more continuous exposure metrics such as "average session length". These must be accompanied by a technical description how a metric like a "session" is defined (e.g., timeout after idle time) [1] (report under item 6a).

subitem not at all important

1 ○

2 ○

3 ○

4 ○

5 ●

essential

Limpar seleção

Does your paper address subitem 17a-i?

Copy and paste relevant sections from the manuscript (include quotes in quotation marks "like this" to indicate direct quotes from your manuscript), or elaborate on this item by providing additional information not in the ms, or briefly explain why the item is not applicable/relevant for your study

Each module has an approximate length of 45 minutes. Women were given the option of pausing the module and resuming the last page visited during subsequent access. Although it was generally recommended to complete one module per week, given the usual competing demands of the postpartum period, women were allowed to complete the program within a maximum period of 8 weeks.

17b) For binary outcomes, presentation of both absolute and relative effect sizes is recommended

Does your paper address CONSORT subitem 17b? *

Copy and paste relevant sections from the manuscript (include quotes in quotation marks "like this" to indicate direct quotes from your manuscript), or elaborate on this item by providing additional information not in the ms, or briefly explain why the item is not applicable/relevant for your study

Not applicable; the adjustment outcomes in this study are continuous.

18) Results of any other analyses performed, including subgroup analyses and adjusted analyses, distinguishing pre-specified from exploratory

Does your paper address CONSORT subitem 18? *

Copy and paste relevant sections from the manuscript (include quotes in quotation marks "like this" to indicate direct quotes from your manuscript), or elaborate on this item by providing additional information not in the ms, or briefly explain why the item is not applicable/relevant for your study

Table 2. Changes in adjustment outcomes and psychological processes over time in the intervention and control groups; Table 3. Two-wave latent change score models estimates.

18-i) Subgroup analysis of comparing only users

A subgroup analysis of comparing only users is not uncommon in ehealth trials, but if done, it must be stressed that this is a self-selected sample and no longer an unbiased sample from a randomized trial (see 16-iii).

subitem not at all important

1 ○

2 ○

3 ○

4 ◉

5 ○

essential

Limpar seleção

Does your paper address subitem 18-i?

Copy and paste relevant sections from the manuscript (include quotes in quotation marks "like this" to indicate direct quotes from your manuscript), or elaborate on this item by providing additional information not in the ms, or briefly explain why the item is not applicable/relevant for your study

This study mainly used intention-to-treat analysis.

19) All important harms or unintended effects in each group

(for specific guidance see CONSORT for harms)

Does your paper address CONSORT subitem 19? *

Copy and paste relevant sections from the manuscript (include quotes in quotation marks "like this" to indicate direct quotes from your manuscript), or elaborate on this item by providing additional information not in the ms, or briefly explain why the item is not applicable/relevant for your study

"...women in the wait-list control group were initially informed about their risk for PPD and instructed to interrupt their participation in the RCT and to seek professional help within the usual care if they felt their health condition was worsening."

19-i) Include privacy breaches, technical problems

Include privacy breaches, technical problems. This does not only include physical "harm" to participants, but also incidents such as perceived or real privacy breaches [1], technical problems, and other unexpected/unintended incidents. "Unintended effects" also includes unintended positive effects [2].

subitem not at all important

- 1 ○
- 2 ○
- 3 ○
- 4 ○
- 5 ◉

essential

Limpar seleção

Does your paper address subitem 19-i?

Copy and paste relevant sections from the manuscript (include quotes in quotation marks "like this" to indicate direct quotes from your manuscript), or elaborate on this item by providing additional information not in the ms, or briefly explain why the item is not applicable/relevant for your study

Not applicable; no privacy breaches or technical problems occurred during this RCT.

19-ii) Include qualitative feedback from participants or observations from staff/researchers

Include qualitative feedback from participants or observations from staff/researchers, if available, on strengths and shortcomings of the application, especially if they point to unintended/unexpected effects or uses. This includes (if available) reasons for why people did or did not use the application as intended by the developers.

subitem not at all important

1 ○

2 ○

3 ○

4 ○

5 ◉

essential

Limpar seleção

Does your paper address subitem 19-ii?

Copy and paste relevant sections from the manuscript (include quotes in quotation marks "like this" to indicate direct quotes from your manuscript), or elaborate on this item by providing additional information not in the ms, or briefly explain why the item is not applicable/relevant for your study

14. Fonseca, A, Pereira, M, Araújo-Pedrosa, A, Gorayeb, R, Ramos, MM, & Canavarro, MC. Be a Mom: Formative evaluation of a web-based psychological intervention to prevent postpartum depression. Cognitive and Behavioral Practice; 2018,25(4):473-495. doi:10.1016/j.cbpra.2018.02.002
15. Fonseca, A, Alves, S, Monteiro, F, Gorayeb, R, Canavarro, MC. “Be a Mom”, a web-based intervention to prevent postpartum depression: Results from a pilot randomized controlled trial. Behavior Therapy; 2020,51(4):616-633. doi:10.1016/j.beth.2019.09.007

DISCUSSION

22) Interpretation consistent with results, balancing benefits and harms, and considering other relevant evidence

NPT: In addition, take into account the choice of the comparator, lack of or partial blinding, and unequal expertise of care providers or centers in each group

22-i) Restate study questions and summarize the answers suggested by the data, starting with primary outcomes and process outcomes (use)

Restate study questions and summarize the answers suggested by the data, starting with primary outcomes and process outcomes (use).

subitem not at all important

1 ○

2 ○

3 ○

4 ○

5 ◉

essential

Limpar seleção

Does your paper address subitem 22-i? *

Copy and paste relevant sections from the manuscript (include quotes in quotation marks "like this" to indicate direct quotes from your manuscript), or elaborate on this item by providing additional information not in the ms, or briefly explain why the item is not applicable/relevant for your study

«"Be a Mom" was found to be uniquely effective in reducing depressive and anxiety symptoms (...) in comparison to the control group where such changes were inexistent or much smaller. (...) All the three psychological processes under study improved significantly (...) in post-treatment assessments: (...) and even if self-compassion increased in both groups, those improvements were considerably greater in the intervention group."

22-ii) Highlight unanswered new questions, suggest future research

Highlight unanswered new questions, suggest future research.

subitem not at all important

1 ○

2 ○

3 ○

4 ○

5 ◉

essential

Limpar seleção

Does your paper address subitem 22-ii?

Copy and paste relevant sections from the manuscript (include quotes in quotation marks "like this" to indicate direct quotes from your manuscript), or elaborate on this item by providing additional information not in the ms, or briefly explain why the item is not applicable/relevant for your study

Cf. Subsection "Conclusions and Future Directions", in the Discussion section.

20) Trial limitations, addressing sources of potential bias, imprecision, and, if relevant, multiplicity of analyses

20-i) Typical limitations in ehealth trials

Typical limitations in ehealth trials: Participants in ehealth trials are rarely blinded. Ehealth trials often look at a multiplicity of outcomes, increasing risk for a Type I error. Discuss biases due to non-use of the intervention/usability issues, biases through informed consent procedures, unexpected events.

subitem not at all important

1 ○

2 ○

3 ○

4 ○

5 ◉

essential

Limpar seleção

Does your paper address subitem 20-i? *

Copy and paste relevant sections from the manuscript (include quotes in quotation marks "like this" to indicate direct quotes from your manuscript), or elaborate on this item by providing additional information not in the ms, or briefly explain why the item is not applicable/relevant for your study

cf. subsection "Limitations" in the Discussion section.

21) Generalisability (external validity, applicability) of the trial findings

NPT: External validity of the trial findings according to the intervention, comparators, patients, and care providers or centers involved in the trial

21-i) Generalizability to other populations

Generalizability to other populations: In particular, discuss generalizability to a general Internet population, outside of a RCT setting, and general patient population, including applicability of the study results for other organizations

subitem not at all important

1 ○

2 ○

3 ○

4 ○

5 ◉

essential

Limpar seleção

Does your paper address subitem 21-i?

Copy and paste relevant sections from the manuscript (include quotes in quotation marks "like this" to indicate direct quotes from your manuscript), or elaborate on this item by providing additional information not in the ms, or briefly explain why the item is not applicable/relevant for your study

Limitations in the generalization of the observed findings are discussed in the subsection "Limitations" (cf. Discussion).

21-ii) Discuss if there were elements in the RCT that would be different in a routine application setting

Discuss if there were elements in the RCT that would be different in a routine application setting (e.g., prompts/reminders, more human involvement, training sessions or other co-interventions) and what impact the omission of these elements could have on use, adoption, or outcomes if the intervention is applied outside of a RCT setting.

subitem not at all important

1 ○

2 ○

3 ○

4 ◉

5 ○

essential

Limpar seleção

Does your paper address subitem 21-ii?

Copy and paste relevant sections from the manuscript (include quotes in quotation marks "like this" to indicate direct quotes from your manuscript), or elaborate on this item by providing additional information not in the ms, or briefly explain why the item is not applicable/relevant for your study

"Moreover, “Be a Mom” has been recently investigated as a universal preventive intervention, also with promising results in the promotion of positive mental health among postpartum women with low risk for PPD [38]."

OTHER INFORMATION

23) Registration number and name of trial registry

Does your paper address CONSORT subitem 23? *

Copy and paste relevant sections from the manuscript (include quotes in quotation marks "like this" to indicate direct quotes from your manuscript), or elaborate on this item by providing additional information not in the ms, or briefly explain why the item is not applicable/relevant for your study

ClinicalTrials.gov (NCT03024645); https://clinicaltrials.gov/ct2/show/NCT03024645

24) Where the full trial protocol can be accessed, if available

Does your paper address CONSORT subitem 24? *

Cite a Multimedia Appendix, other reference, or copy and paste relevant sections from the manuscript (include quotes in quotation marks "like this" to indicate direct quotes from your manuscript), or elaborate on this item by providing additional information not in the ms, or briefly explain why the item is not applicable/relevant for your study

14. Fonseca, A, Pereira, M, Araújo-Pedrosa, A, Gorayeb, R, Ramos, MM, & Canavarro, MC. Be a Mom: Formative evaluation of a web-based psychological intervention to prevent postpartum depression. Cognitive and Behavioral Practice; 2018,25(4):473-495. doi:10.1016/j.cbpra.2018.02.002
Data availability statement: The data that support the findings of this study are available from the corresponding author, upon reasonable request.

25) Sources of funding and other support (such as supply of drugs), role of funders

Does your paper address CONSORT subitem 25? *

Copy and paste relevant sections from the manuscript (include quotes in quotation marks "like this" to indicate direct quotes from your manuscript), or elaborate on this item by providing additional information not in the ms, or briefly explain why the item is not applicable/relevant for your study

See "Acknowledgements" section.

X27) Conflicts of Interest (not a CONSORT item)

X27-i) State the relation of the study team towards the system being evaluated

In addition to the usual declaration of interests (financial or otherwise), also state the relation of the study team towards the system being evaluated, i.e., state if the authors/evaluators are distinct from or identical with the developers/sponsors of the intervention.

subitem not at all important

1 ○

2 ○

3 ○

4 ○

5 ○

essential

Does your paper address subitem X27-i?

Copy and paste relevant sections from the manuscript (include quotes in quotation marks "like this" to indicate direct quotes from your manuscript), or elaborate on this item by providing additional information not in the ms, or briefly explain why the item is not applicable/relevant for your study

Right in the Introduction section, it stands clear that the authors were the developers of this software (e.g., reference 14). If required, please include the following Declaration of interest: "All the authors participated in the development of the intervention."

About the CONSORT EHEALTH checklist

As a result of using this checklist, did you make changes in your manuscript? *

○ yes, major changes

○ yes, minor changes

◉ no

What were the most important changes you made as a result of using this checklist?

Suggestion: «Declaration of interest: "All the authors participated in the development of the intervention."»

How much time did you spend on going through the checklist INCLUDING making changes in your manuscript *

Taken altogether, between 3-4 working hours.

As a result of using this checklist, do you think your manuscript has improved? *

- ( ) yes
- ( ) no
- (•) Outra: We have carefully adopted this checklist when preparing our manuscr

Would you like to become involved in the CONSORT EHEALTH group?

This would involve for example becoming involved in participating in a workshop and writing an "Explanation and Elaboration" document

- (•) yes
- ( ) no
- ( ) Outra:

Limpar seleção

Any other comments or questions on CONSORT EHEALTH

This initiative is very much appreciated towards zealous research.

STOP - Save this form as PDF before you click submit

To generate a record that you filled in this form, we recommend to generate a PDF of this page (on a Mac, simply select "print" and then select "print as PDF") before you submit it.

When you submit your (revised) paper to JMIR, please upload the PDF as supplementary file.

Don't worry if some text in the textboxes is cut off, as we still have the complete information in our database. Thank you!

Final step: Click submit !

Click submit so we have your answers in our database!

Enviar Limpar formulário

Nunca envie palavras-passe através dos Google Forms.

Este conteúdo não foi criado nem aprovado pela Google. Denunciar abuso - Termos de Utilização - Política de privacidade

Google Formulários
