# Supplementary material for: The Efficacy of Be a Mom, a Web-Based Intervention to Prevent Postpartum Depression: Examining Mechanisms of Change in a Randomized Controlled Trial
Source: JMIR Ment Health. 2023 Mar 17;10:e39253. doi: 10.2196/39253 (PMC10139682; doi:10.2196/39253)
Supplement: Multimedia Appendix 2 [file mental_v10i1e39253_app2.docx]

**Table S1.** Changes in adjustment outcomes and psychological processes over time in the intervention and control groups: Descriptives and Univariate Latent Change Scores estimates.

|  | Descriptives | | | |  | |  |  | |  | | |  | | Univariate Latent Change Scores (LCS) | | | | | | | |  |
| --- | --- | --- | --- | --- | --- | --- | --- | --- | --- | --- | --- | --- | --- | --- | --- | --- | --- | --- | --- | --- | --- | --- | --- |
|  | Intervention Group | | Control Group | |  | |  | Intervention Group | | | | | |  | |  | |  | | Control Group | | |  |
|  | T0  (*n*=542)  *M (SD)* | T1  (*n*=267)  *M (SD)* | T0  *(n=511)*  *M (SD)* | T1  (*n*=445)  *M (SD)* | μ_Δ_  *B(SE)* | *P* | | | σ^2^_Δ_  *B(SE)* | | *P* | σ1  *B(SE)* | | *P* | | μ_Δ_  *B(SE)* | *P* | | σ^2^_Δ_  *B(SE)* | | *P* | σ1  *B(SE)* | *P* |
|  |  |  |  |  |  |  | | |  | |  |  | |  | |  |  | |  | |  |  |  |
| EPDS | 10.99 (5.14) | 8.75 (4.53) | 11.73 (4.81) | 10.78 (5.06) | -2.27 (0.24) | <.001 | | | 20.11 (1.71) | | <.001 | -12.97 (1.43) | | <.001 | | -0.79 (0.19) | <.001 | | 16.67 (1.12) | | <.001 | -6.92 (0.99) | <.001 |
| HADS | 7.77 (4.13) | 6.57 (3.93) | 8.40 (4.21) | 8.24 (4.21) | -1.36 (0.20) | <.001 | | | 13.53 (1.16) | | <.001 | -7.53 (0.95) | | <.001 | | 0.00 (0.16) | .993 | | 12.61 (0.85) | | .001 | -.62 (0.77) | <.001 |
| DERS | 42.28 (13.29) | 39.47 (13.07) | 44.26 (13.50) | 42.77 (13.87) | -2.87 (0.71) | <.001 | | | 135.62 (12.59) | | <.001 | -69.88 (10.26) | | <.001 | | -0.82 (0.48) | .091 | | 103.71 (7.22) | | <.001 | -44.13 (7.15) | .001 |
| SCS | 35.80 (8.95) | 34.93 (8.64) | 38.17 (8.68) | 35.65 (9.41) | 2.90 (0.46) | <.001 | | | 57.25 (5.21) | | <.001 | -32.77 (4.25) | | <.001 | | 0.65 (0.27) | .015 | | 29.35 (2.06) | | <.001 | -8.96 (2.31) | <.001 |
| CompACT | 58.56 (14.33) | 61.30 (15.53) | 56.36 (14.47) | 57.25 (15.11) | 2.82 (0.83) | .001 | | | 185.18 (16.58) | | <.001 | -79.15 (12.04) | | <.001 | | 0.37 (0.48) | .442 | | 103.41 (7.07) | | <.001 | -45.51 (7.26) | <.001 |
|  |  |  |  |  |  |  | | |  | |  |  | |  | |  |  | |  | |  |  |  |

*Note.* T0 = baseline assessment; T1 = post-intervention assessment; μ_Δ_ = mean/intercept of the latent change factor; σ^2^_Δ =_ variance of the latent change factor. EPDS = Depressive symptoms. HADS-A = Anxiety symptoms. DERS = Difficulties in Emotion Regulation. SCS = Self-Compassion. CompACT = Psychological Flexibility. The LCS estimates presented correspond to the unconstrained model (where LCS parameters were free to vary across groups, except for means at baseline, which were constrained to be equal across groups). Unstandardized estimates are presented.
